# Supplementary material for: COS-PPA: protocol to develop a core outcome set for primary progressive aphasia
Source: BMJ Open. 2024 May 6;14(5):e078714. doi: 10.1136/bmjopen-2023-078714 (PMC11086495; doi:10.1136/bmjopen-2023-078714)
Supplement: Supplementary data [file bmjopen-2023-078714supp001.pdf]

IMPROVING

PRIMARY PROGRESSIVE APHASIA: DEVELOPMENT OF A CORE OUTCOME SET

NOMINAL GROUP TECHNIQUE (NGT)

MANUAL

CHIEF INVESTIGATOR:

PI: DR ANNA VOLKMER

LANGUAGE AND COGNITION, UCL

ADAPTED WITH PERMISSION FROM DR SARAH WALLACE

Volkmer A, *et al. BMJ Open* 2024; 14:e078714. doi: 10.1136/bmjopen-2023-078714

HOW TO USE THIS MANUAL

This manual contains the information that you will need to run your nominal group/s.

Throughout the manual you will see the following symbols:

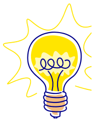

The 'light bulb' symbol will alert you to tips or extra information.

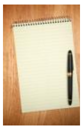

The 'paper and pen' symbol indicates that there is a relevant template in the appendix. You **may need to translate these documents or modify them** to reflect your language/ location or details specific to your site.

Any questions or comments about this manual or study can be directed to

Anna Volkmer

IMPROVING

PRIMARY PROGRESSIVE APHASIA: DEVELOPMENT OF A CORE OUTCOME SET

TABLE OF CONTENTS

HOW TO USE THIS MANUAL **2**

1. EXECUTIVE SUMMARY ..... ERROR! BOOKMARK NOT DEFINED.

2. PROJECT OVERVIEW **5**

2.1 PROJECT AIM 5

2.2 BACKGROUND 5

2.3 PROJECT OVERVIEW 6

2.4 STUDY 1A: NOMINAL GROUP TECHNIQUE (NGT) 6

2.4.1 Objectives *6*

2.4.2 NGT overview *7*

2.4.3 Stages of the NGT *7*

2.4.4 Benefits of the NGT *8*

3. ETHICAL APPROVAL **8**

4. PARTICIPANTS **8**

4.1 ELIGIBILITY AND SAMPLING 8

4.2 GROUP COMPOSITION 10

5. RECRUITMENT **10**

6. VENUE AND RESOURCES **11**

6.1 VENUE 11

6.2 EQUIPMENT 11

7. PROCEDURES FOR RUNNING NOMINAL GROUPS **12**

9. PARTICIPANT FOLLOW-UP ..... ERROR! BOOKMARK NOT DEFINED.

10. CONTACT DETAILS **17**

11. APPENDIX ..... ERROR! BOOKMARK NOT DEFINED.

B. Slides and Nominal question ..... **Error! Bookmark not defined.**

C. Participant information sheet ..... **Error! Bookmark not defined.**

E. Participant consent form ..... **Error! Bookmark not defined.**

G. Participant demographics ..... **Error! Bookmark not defined.**

H. participant response sheet ..... **Error! Bookmark not defined.**

12. REFERENCES **17**

IMPROVING

PRIMARY PROGRESSIVE APHASIA: DEVELOPMENT OF A CORE OUTCOME SET

1. PROJECT OVERVIEW

1.1 PROJECT AIM

To develop a core outcome set for research on intervention with people with PPA.

1.2 BACKGROUND

There is currently no curative treatment for Primary Progressive Aphasia (PPA), a language led dementia that progresses inexorably over time. Symptomatic pharmacological therapies have also not shown any evidence of effectiveness. Speech and language therapists and neuroscientists across the world have, however, worked for many years on tailored programmes for such people with PPA, and multiple interventions have emerged. Yet, research examining the effectiveness of these lack rigour, with small sample sizes and a lack of consistency in outcome measures posing limitations to the generalisability of the work. In this international cross-disciplinary collaboration we propose the development of a core set of outcome measures for researchers in the field of PPA interventions. This is extremely important in allowing this field of intervention research to develop, improving the rigour and impact of the work being undertaken. This will provide benefits for individuals with PPA worldwide, increasing access to interventions that can maintain communication, relationships and indepdenendence.

The COMET (Core Outcome Measures in Effectiveness Trials) initiative seeks to connect people interested in the development of core outcome sets. The COMET website houses a database (see http://www.comet-initiative.org) which currently contains 196 references of planned, ongoing and completed work on core outcome sets. OMERACT (Outcome Measures for Rheumatology Clinical Trials) is perhaps the best known initiative for the development of core outcome sets. OMERACT has used an iterative consensus process to develop a core outcome set for use in rheumatology clinical trials. It has a great focus on stakeholder consultation, with consumer, research, clinical and industry stakeholders participating in development process (Kirwan et al., 2003; Kvien & Heiberg, 2003; Quest et al., 2003). There is growing acknowledgement of the benefits of core outcome sets for research. Core sets have been developed or are being developed in over 50 fields including chronic pain (Dworkin et

al., 2005; McGrath et al., 2008; Turk et al., 2008), systemic sclerosis (Khanna, 2008; Khanna et al., 2008), childhood asthma (Sinha et al., 2012) and Eczema (Schmitt, Langan, Stamm, Williams, & Harmonizing Outcome Measurements in Eczema Delphi, 2011).

1.3 PROJECT OVERVIEW

This study will comprise a Nominal Group Technique (NGT) protocol previously devised by our collaborator, Dr Sarah Wallace, modifed to meet the needs of people with PPA. We will lead a team of international collaborators to hold meetings in countries identified through our networks of collaborators. Meetings will be held remotely where required by COVID-19 restrictions.

Participants and recruitment: Stakeholders will be purposivefully invited from third sector organisations, clinical and research networks, to represent people with PPA, their families, clinical speech and language therapists, neurologists and neuropsychologists. Meetings with people with PPA will be held separately from their family members and will therefore not exceed more than 4-6 participants to ensure the facilitator will be able to support participants' communication needs.

Analysis: Data will be analysed using aggregation methods consistent with NGT methodology.

1.4 STUDY 1A: NOMINAL GROUP TECHNIQUE (NGT)

1.4.1 OBJECTIVES

1. To identify outcomes of importance to people with PPA and their family and friends from an international sample.

2. To prioritise outcomes of importance to people with PPA and their family and friends from an international sample.

3. To categorise outcomes of importance into outcome domains (using World Health Organization (WHO) International Classification of Functioning, Disability and Health (ICF) domains (World Health Organization., 2001)).

4. To examine similarities and differences in outcomes of importance across stakeholder and country groups.

5. To determine which outcome domains have the highest representation within and across stakeholder and country groups.

6. To identify the ultimate desired outcome of speech and language therapy interventions according to participants with PPA and their family and friends.

1.4.2 NGT OVERVIEW

NGT is a structured group decision-making technique. In this technique a small group of participants are asked to respond to a question posed by a group facilitator. Participants are then asked to rank or prioritise these responses. The individual votes are then tallied to identify the ideas that are rated highest by the group as a whole. The NGT process encourages the participation of all group members and results in a set of prioritised responses (Delbecq et al., 1975). It is widely recognised as an effective method of gaining group consensus (Allen, Dyas, & Jones, 2004; Harvey & Holmes, 2012).

1.4.3 STAGES OF THE NGT

The NGT is divided into the following stages:

- Welcome and introduction: Purpose of session, rules and structure.
- Stage 1: Individual responses are collected.
- Stage 2: Individual responses are clarified and similar responses are grouped.
- Stage 3: Participants rank their top three responses in terms of personal importance.
- Responses are calculated and shared with the group.

- Thanks and close.

1.4.4 BENEFITS OF THE NGT

NGT has been previously used in the development of core outcome sets as a means of achieving consensus on outcomes, outcome domains and outcome instruments for inclusion in core sets (Douglas et al., 2009; Heiligenhaus et al., 2012; Khanna et al., 2008; Lamb et al., 2005).

NGT is suited to use with people with communication disability as it inherently supports communication through a structured 'round-robin' process of idea presentation which allows equal participation. The technique also encourages 'hitchhiking' (the stimulation of ideas in response to other group member responses) which increases opportunities for participation (Delbecq et al., 1975).

The technique has been used successfully with people with aphasia (Dorze, Julien, Brassard, Durocher, & Boivin, 1994; Garcia, Laroche, & Barrette, 2002; Lomas et al., 1989; Lomas, Pickard, & Mohide, 1987) and people with traumatic brain injury and associated communication disability (Larkins et al., 2004).

NGT is a cost and time effective method of data collection as the results are immediately available and no transcription is required.

2. ETHICAL APPROVAL

Ethical approval in the UK is part of the IMPACT study, UCL.

3. PARTICIPANTS

The NGT study has two participant groups: 1) people with PPA and 2) the family and friends of people with PPA and 3) bereaved family and friends of people with PPA.

3.1 ELIGIBILITY AND SAMPLING

People with PPA

Inclusion criteria:

Volkmer A, et al. BMJ Open 2024; 14:e078714. doi: 10.1136/bmjopen-2023-078714

- Aged 18 years or over.
- Diagnosis of PPA
- Ability to participate in the nominal group technique (NGT) process (as judged by an SLT).
- Living in the community.

Exclusion criteria:

- Comorbid significant health or mental health impairments (e.g. stroke or severe depression)

Sampling: Maximum variation sampling.

Sampling variables:

- Age
  - <100 years
  - >18 years
- Variant
  - Logopenic
  - Semantic
  - Non-fluent
- Gender
  - Male
  - Female
- World region
  - Africa
  - Asia
  - Australia
  - The Americas
  - Europe

Family and friends of people with PPA

Selection criteria:

- As above, except not diagnosed with PPA

Sampling: Convenience sampling

Volkmer A, *et al. BMJ Open* 2024; 14:e078714. doi: 10.1136/bmjopen-2023-078714

3.2 GROUP COMPOSITION

There should be a maximum 5 people per nominal group. Site co-ordinators should aim for at least one group of people with PPA and one group of family and friends.

For groups of people with PPA, try to ensure that as many of the following variables as possible are represented:

Gender	variant	Time post-onset	Age
Male	semantic	< 1 year	< 65 years
Female	logopenic	> 1 year	> 65 years
	Non-fluent		

4. RECRUITMENT

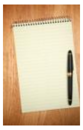

It is suggested that the onsite speech and language therapist/pathologist should recruit people with PPA and their family and friends through local avenues, such as email discussion groups, newsletters and community groups.

Interested participants should be provided with a more detailed information sheet (to be produced locally).

People with PPA who agree to participate in this study should also be asked to nominate a family member or friend to participate.

Separate nominal groups should be held for people with PPA and family/friend groups.

One or more groups may be held for each participant group. Each nominal group should have a maximum of 5 participants.

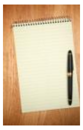

Demographic details should be recorded for each participant comprising diagnosis, time since onset, gender, age.

5. VENUE AND RESOURCES

5.1 VENUE

The nominal groups will be run online on zoom. Prior to facilitating the group the following should be clarified.

- Are clients able to access zoom with support as required?
- Can keywords can be written/typed to help facilitate the comprehension of people with PPA?

5.2 EQUIPMENT

To run your nominal group you will need:

- Pens and paper (for participants)
- Powerpoint slides pre-prepared with key questions/ headings
- Communication supports for participants e.g. communication (picture) boards
- Video recording facilities via zoom
- Some may need help from carers/ family members – to assist with collecting demographic details and recording participant responses.

6. PROCEDURES FOR RUNNING NOMINAL GROUPS

Each nominal group should have a maximum of 5 participants. People with PPA may have family member/friend present to support them if they desire. You may wish to have additional helpers to assist with collecting demographic information, write field notes and to help people with PPA to communicate their responses.

BEFORE THE DAY OF THE GROUP

Assign each participant a number

Each participant should be assigned a participant number using the following format:

**Country**	**Stakeholder group**	**Group number**	**Number**
Australia – AU<br>Canada – CA<br>United States – US<br>Israel– IL<br>Singapore – SP<br>India- In<br>Chile- Ch<br>Germany- Gr<br>United Kingdon- UK<br>Norway-Nw<br>Turkey- Tk<br>Iceland- IC	People with PPA – PWPPA<br>Family and friends - FF	If you have more than 1 nominal group for each stakeholder type, use a group number	Give each participant a number

e.g. AU-PWA-1-1

AU-PWA-1-2

ON THE DAY OF THE GROUP

Before the participants arrive

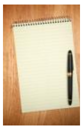

Consent will be sought prior to the group.

Meet prior to the NGT meeting/Email/ post the information sheet, consent form, question and response sheet and demographic information form for each participant.

Introduction (10 minutes)

Welcome the participants and provide a brief explanation of the purpose of the study and an outline of the procedure for the group using the following scripts. You may modify these scripts if necessary, however we are aiming for as much consistency across sites as possible. When presenting the introduction to people with PPA write key words on the whiteboard to maximise communicative access to the information.

Introduction Script – People with PPA

Thank you for coming today.

We are doing this study because we want to learn more about the outcomes (results from speech and language therapy) that are important to people with PPA.

We hope that this will help to improve PPA research and PPA services.

We are using a technique called a 'nominal group technique' to find out what is important to you. It allows everyone to contribute equally.

I will ask the group a question. Everyone will have time to think about their answers. We will then take turns to share our answers. Your answers don't have to be the ones you first thought of. If you think of something else you can add it. We will write the answers on the whiteboard. We will group similar answers together. You will have a chance to explain your responses. Then I will ask you to pick the three most important answers to you. We will help you to write the answers down on your sheet.

The information that you give today will be kept confidential and safe. The results may be published in a journal. Your name will not be used.

You will need to sign a consent form. It is your choice to participate in this study. It will take approximately an hour. You can stop anytime.

We will be video recording the group. This will be used check the information we collect. Are there any questions?

Introduction Script – Family and friends of people with PPA

Thank you for coming today.

This study aims to find out what outcomes are important to family and friends of people with PPA. We want to develop a set of tools to measure change following speech and language therapy. This is called a core outcome set. This will help us to improve PPA research. We hope it will also improve the services received by people living with PPA.

We will also be asking people with PPA, SLTs, policy makers and researchers what is important to them.

We are using a technique called a 'nominal group technique' to find out what is important to you. We are using this technique as it has been shown to allow everyone to contribute equally.

I will ask the group a question. Everyone will have time to think about their answers. We will then take turns to share our answers. Your answers don't have to be the ones you originally thought of. If you think of something else you can add it. We will write the answers on the whiteboard. We will group similar answers together. You will have a chance to explain or clarify your responses. Then I will ask you to pick the three most important answers to you and write them on your response sheet.

The information that you give today will be kept confidential and stored securely. The results may be published in a journal, but your name will not be used.

You will need to sign a consent form. It is your choice to participate in this study. You can stop anytime.

We will be video-recording the group. This will be used for data checking. It will take approximately 1 hour. Are there any questions?

Stage 1: Individual responses (25 minutes)

Start videoing. Inform participants that the video has been turned on and is now recording.

Read the following information to the group and present the nominal question verbally and in writing (write the question in large print on the whiteboard).

People with PPA	What would you most like to change about your communication and the way PPA affects your life?
Family and friends of people with PPA	What would you most like to change about your family member/friend's communication and the way PPA affects your life?

Allow a period of 5-10 minutes of/for quiet reflection. Ask group members to write down (if they are able) or think about as many responses as possible. If you have additional helpers in the room, they may assist people with PPA to write down their responses.

Give each group member the opportunity to verbally offer one response at a time in rounds, allowing all participants the opportunity to contribute their answers. Write responses onto a slide, which you are screen sharing with participants.

Encourage 'hitchhiking' – i.e. encourage group members to expand on the responses of other group members. Responses do not need to come from the individual's original list.

Stage 2: Clarification and consolidation (20 minutes)

Read through each response. Allow participants to explain or clarify their responses.

Group similar responses together. Duplicates may be combined or deleted.

Stage 3: Ranking responses (15 minutes)

Ask the participants to reflect on their own feelings and beliefs.

Ask participants to select the three outcomes that are most important to them from the group list.

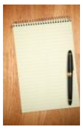

Ask participants to rank their top three most important outcomes, in order of personal importance. Enter this on the relevant slide in ranke order. Mark the column with the participant number.

AFTER THE GROUP

Thank participants.

Collate the final responses from each participant.

Note the amount of time that the session took.

7. DATA MANAGEMENT

The data will be stored safely on an encrypted laptop.

8. CONTACT DETAILS

Please direct any questions or correspondence to whomever is facilitating the group localy, or alternative Dr Anna Volkmer a.volkmer.15@ucl.ac.uk.

9. REFERENCES

Allen, J., Dyas, J., & Jones, M. (2004). Building consensus in health care: a guide to using the nominal group technique. [Research Support, Non-U.S. Gov't]. *Br J Community Nurs, 9*(3), 110-114.

Dorze, G. L., Julien, M., Brassard, C., Durocher, J., & Boivin, G. (1994). An analysis of the communication of adult residents of a long-term care hospital as perceived by their caregivers. *International Journal of Language & Communication Disorders, 29*(3), 241-268. doi: doi:10.3109/13682829409111610

Douglas, R. S., Tsirbas, A., Gordon, M., Lee, D., Khadavi, N., Garneau, H. C., . . . International Thyroid Eye Disease, S. (2009). Development of criteria for evaluating clinical response in thyroid eye disease using a modified Delphi technique. [Research Support, N.I.H., Extramural

Research Support, Non-U.S. Gov't]. *Arch Ophthalmol, 127*(9), 1155-1160. doi: 10.1001/archophthalmol.2009.232

Garcia, L. J., Laroche, C., & Barrette, J. (2002). Work integration issues go beyond the nature of the communication disorder. *Journal of Communication Disorders, 35*(2), 187-211. doi: http://dx.doi.org/10.1016/S0021-9924(02)00064-3

Harvey, N., & Holmes, C. A. (2012). Nominal group technique: an effective method for obtaining group consensus. [Research Support, Non-U.S. Gov't]. *Int J Nurs Pract, 18*(2), 188-194. doi: 10.1111/j.1440-172X.2012.02017.x

Heiligenhaus, A., Foeldvari, I., Edelsten, C., Smith, J. R., Saurenmann, R. K., Bodaghi, B., . . . Multinational Interdisciplinary Working Group for Uveitis in, C. (2012). Proposed

outcome measures for prospective clinical trials in juvenile idiopathic arthritis-associated uveitis: a consensus effort from the multinational interdisciplinary working group for uveitis in childhood. [Consensus Development Conference

Practice Guideline

Research Support, Non-U.S. Gov't

Review]. *Arthritis Care Res (Hoboken), 64*(9), 1365-1372. doi: 10.1002/acr.21674

Khanna, D., Lovell, D. J., Giannini, E., Clements, P. J., Merkel, P. A., Seibold, J. R., . . . Scleroderma Clinical Trials Consortium, c.-a. (2008). Development of a provisional core set of response measures for clinical trials of systemic sclerosis. [Research Support, N.I.H., Extramural

Research Support, Non-U.S. Gov't]. *Ann Rheum Dis, 67*(5), 703-709. doi: 10.1136/ard.2007.078923

Lamb, S. E., Jorstad-Stein, E. C., Hauer, K., Becker, C., Prevention of Falls Network, E., & Outcomes Consensus, G. (2005). Development of a common outcome data set for fall injury prevention trials: the Prevention of Falls Network Europe consensus. [Research Support, Non-U.S. Gov't]. *J Am Geriatr Soc, 53*(9), 1618-1622. doi: 10.1111/j.1532-5415.2005.53455.x

Lomas, J., Pickard, L., Bester, S., Elbard, H., Finlayson, A., & Zoghaib, C. (1989). The Communicative Effectiveness Index: Development and Psychometric Evaluation of a Functional Communication Measure for Adult Aphasia. *J Speech Hear Disord, 54*(1), 113-124.

Lomas, J., Pickard, L., & Mohide, A. (1987). Patient versus Clinician Item Generation for Quality-of-Life Measures: The Case of Language-Disabled Adults. *Medical Care, 25*(8), 764-769. doi: 10.2307/3765712
