# Supplementary material for: COS-PPA: protocol to develop a core outcome set for primary progressive aphasia
Source: BMJ Open. 2024 May 6;14(5):e078714. doi: 10.1136/bmjopen-2023-078714 (PMC11086495; doi:10.1136/bmjopen-2023-078714)
Supplement: Author's manuscript [file bmjopen-2023-078714.draft_revisions.pdf]

BMJ Open

BMJ Open is committed to open peer review. As part of this commitment we make the peer review history of every article we publish publicly available.

When an article is published we post the peer reviewers' comments and the authors' responses online. We also post the versions of the paper that were used during peer review. These are the versions that the peer review comments apply to.

The versions of the paper that follow are the versions that were submitted during the peer review process. They are not the versions of record or the final published versions. They should not be cited or distributed as the published version of this manuscript.

BMJ Open is an open access journal and the full, final, typeset and author-corrected version of record of the manuscript is available on our site with no access controls, subscription charges or pay-per-view fees (http://bmjopen.bmj.com).

If you have any questions on BMJ Open's open peer review process please email info.bmjopen@bmj.com

BMJ Open

COS- PPA: Protocol to develop a core outcome set for Primary Progressive Aphasia

Journal:	*BMJ Open*
Manuscript ID	bmjopen-2023-078714
Article Type:	Protocol
Date Submitted by the Author:	09-Aug-2023
Complete List of Authors:	Volkmer, Anna; University College London, Department of Psychology and Language Sciences<br>Copland, David; The University of Queensland - Saint Lucia Campus, School of Health and Rehabilitation Sciences; The University of Queensland, Queensland Aphasia Research Centre<br>Henry, Maya L.; The University of Texas at Austin<br>Varley, Rosemary; University College London, Department of Psychology and Language Sciences<br>Wallace, Sarah J.; The University of Queensland, School of health and rehabilitation sciences; The University of Queensland<br>Hardy, Chris ; University College London, Dementia Research Centre
Keywords:	Dementia, Speech pathology < OTOLARYNGOLOGY, Patient Reported Outcome Measures

SCHOLARONE™
Manuscripts

Title:

COS- PPA: Protocol to develop a core outcome set for Primary Progressive Aphasia

Volkmer, Anna.[1] (a.volkmer.15@ucl.ac.uk), Copland, David.[3,4], Henry, Maya L.[6], Warren, Jason D. [2], Varley, Rosemary[1]., Wallace, Sarah J.[3,4] Hardy, Chris J.D.[2]

Affiliations:

1. Department of Psychology and Language Sciences, University College London, London, UK.
2. Dementia Research Centre, Department of Neurodegenerative Disease, UCL Queen Square Institute of, Neurology, University College London, London, UK
3. Queensland Aphasia Research Centre, School of Health and Rehabilitation Sciences, The University of Queensland, Brisbane, Australia
4. Surgical Treatment and Rehabilitation Service (STARS) Education and Research Alliance, The University of Queensland and Metro North Health, Queensland, Australia
5. Departments of Speech, Language, and Hearing Sciences and Neurology, University of Texas, Austin, USA.

Contact information:
a.volkmer.15@ucl.ac.uk
University College London
Chandler House
2 Wakefield Street
London
WC1N 1PF

For peer review only

Abstract:

Background:

The term Primary Progressive Aphasia (PPA) describes a group of language-led dementias. Disease-modifying treatments for PPA are currently lacking, though a number of symptomatic interventions have been developed in recent years. Unfortunately, studies exploring the effectiveness of these interventions have used a variety of different outcome measures, limiting comparability. Typically, these measures do not necessarily assess what is important to key stakeholders. Existing core outcome sets (COS) for dementia and non-progressive aphasia do not meet the needs of people with primary progressive aphasia, highlighting a need to develop a specific core outcome set for PPA.

Methods: This protocol describes a three-stage study to identify a COS for Primary Progressive Aphasia interventions in research and clinical practice. The Stage 1 systematic review will identify existing speech, language and communication measures used to examine the effectiveness of interventions for PPA in the research literature. Employing a Nominal Group Technique, Stage 2 will identify the most important outcomes to key stakeholders internationally, including people with PPA and their families. The data collected in Stages 1 and 2 will be jointly analysed with the project PPI group and will inform the Stage 3 modified Delphi consensus study to identify a core outcome measurement set for PPA amongst a range of research disciplines undertaking intervention studies for people with PPA.

Discussion: The development of a core outcome set for PPA will inform the design of intervention studies in the field of PPA. Measuring outcomes that are meaningful to key stakeholders will ensure interventions demonstrate relevant outcomes that address the needs of this population and will support clinicians to make more informed decisions in clinical practice.

Trial registration: Registered with COMET March 2021: https://www.comet-initiative.org/Studies/Details/1871

Keywords:

Primary Progressive Aphasia

Core Outcome Set

For peer review only - http://bmjopen.bmj.com/site/about/guidelines.xhtml

Speech and Language Therapy

Interventions

Background:

Primary Progressive Aphasia (PPA) is a term used to describe a group of speech and language-led dementias that progress inexorably over time [1,2,3]. Three major PPA syndromes have been identified. Semantic variant PPA (svPPA) presents with difficulties in producing and understanding words and is associated with frontotemporal lobar degeneration. Nonfluent variant (nfvPPA) is characterised by a motor-speech disorder called speech apraxia, which presents as groping effortful speech, and/or difficulties in using grammar (agrammatism) and is also associated with frontotemporal lobar degeneration. PPA Apraxia of Speech (PPA AOS), describes a 'purer' form of nfvPPA, and can be further fractioned into phonetic or prosodic subtypes, but is generally considered part of the broader nfvPPA syndrome . Finally, Logopenic variant PPA (lvPPA) results in difficulties in word retrieval and phonological working memory and is chiefly associated with Alzheimer's pathology [3].

There is currently no disease-modifying treatment for any form of PPA and symptomatic pharmacological therapies have also not shown evidence of effectiveness across all PPA variants. Speech and language therapists, psychologists and neuroscientists across the world have however, developed multiple tailored interventions for people with PPA [4,5,6,7,8]. Yet, research examining the effectiveness of these interventions has generally focused on case studies, and cohorts with sample sizes. Only one randomised controlled trial has been reported in the intervention literature to date [9]. Importantly, there has also been huge variability in outcome measures used across all these studies [4,8].

Given the rarity and clinical heterogeneity of this disease, being able to group data would strengthen outcomes and knowledge about generalisability of interventions for people with PPA. Similarly, comparing data can enhance evidence-based clinical decision making and service development [10]. Ensuring that measures are meaningful to key stakeholders, including people with the disease, their families and relevant health care professionals will be vital to this process [11]. The huge variability in outcomes used within the PPA intervention

For peer review only - http://bmjopen.bmj.com/site/about/guidelines.xhtml

literature places limitations on aggregation of outcome data, comparisons across studies and ultimately the delivery of appropriate clinical services.

A Core Outcome Set (COS) is an agreed set of outcomes that are measured and reported in intervention studies related to a particular health condition [11]. The main reason to develop a Core Outcome Set is to allow for comparison across similar trials, standardise reporting to reduce selective and/or biased reporting, and make results more relevant [12]. There has been previous work undertaken to develop a COS for use in the evaluation of non-pharmacological interventions for all-cause dementia which identified a long list of 54 items [13,14]. Though this generic list included communication, the list was not salient to interventions with the similar objectives (it encompassed a wide variety) and did not reflect the views of stakeholders internationally. The authors of the review also acknowledge that the constructs identified in this work are broad and overlapping and therefore lacking specificity [15] in terms of the measures to be used.

Work has been undertaken to identify a COS for stroke aphasia. The Research Outcome Measurement in Aphasia (ROMA)-COS identified five essential outcome constructs and appropriate measurement instruments that address each domain [16,17] including: language, communication, patient-reported satisfaction with treatment and impact of treatment, emotional wellbeing, and quality of life. Importantly, the scope of the ROMA-COS focused on rehabilitation of non-progressive aphasia. Given our understanding of the unique needs of people with PPA [18], there is a need for a specific COS, that details key measures addressing the needs of people with PPA. Given the heterogeneity of this disease, this should include consideration of the value of different outcomes for different PPA syndromes [19]. There is also an urgent need to improve diversity of access to care and supports in PPA, socioeconomically, linguistically and culturally. To address potential barriers to implementation of measures, incorporating an international and cross-disciplinary stakeholder perspective will be essential.

The aim of this international cross-disciplinary collaboration is to develop a core set of outcome measures for researchers in the field of PPA interventions. Identifying 'what' and 'how' best to measure outcomes will inform future developments in PPA intervention research, as well as improving the impact of the work being undertaken. This, in turn, will benefit individuals with

PPA by increasing access to evidence-based interventions. Thus, the objectives of this study are:

- To extract the speech, language and communication measures used to examine the effectiveness of interventions for PPA to date in the research literature.
- To identify the most important outcomes to key stakeholders, including people with PPA, their families and speech and language therapists.
- To identify outcomes that address the needs of people with different PPA variants.
- To achieve consensus on a COS for PPA (the PPA-COS) amongst a range of research disciplines undertaking intervention studies for people with PPA.

Scope:

1. The health condition and population covered by this COS:
   The PPA-COS covers people with PPA (inclusive of PPAOS), as defined by the current internationally accepted diagnostic criteria [1].

2. The interventions covered by this COS:
   The PPA-COS covers intervention research that aims to affect all domains of speech, language and communication, including communication related quality of life and wellbeing, for people living with PPA and their communication partners.

3. The settings covered by this COS:
   The setting covered by this COS-PPA covers intervention research and clinical delivery of interventions to people with PPA internationally, with the goal of capturing key stakeholders across all major World Health Organisation's regions of the world [20] who speak and work with people with PPA in a variety of languages. We anticipate that there may be issues with measures that have not yet been translated into languages other than English and will consider the implications of this during this process.

Methods:

The 11 minimum standards outlined in the COS-Standards for Development Recommendations (COS-STAD) [12] informed the development of this protocol to ensure the scope, stakeholders and consensus processes were all addressed in line with these recommendations. The COS-

Standardised Protocol (COS-STAP) [21] Protocol Items consisting of a checklist of 13 items and accompanying COS-STAP Explanation and Elaboration (E+E) [21] document were also consulted to ensure the COS-PPA development plans, as well as stakeholder involvement and consensus processes, were adequately addressed (see supplementary information for completed COS-STAP reporting checklist). The COS-PPA was registered on the COMET website in March 2021: https://www.comet-initiative.org/Studies/Details/1871. In addition, the Stage 1 systematic review was preregistered on PROSPERO in December 2022: CRD42022367565.

Stakeholders:

Members of the research team

1. *Patient and Public Involvement (PPI)*

   The development of this study proposal was informed by PPI work undertaken during the Better Conversations with PPA (BCPPA) randomised controlled pilot study [9]. PPI collaborators identified that measurement tools needed to reflect what was important to people living with PPA [22]. The current COS-PPA study is overseen by the BCPPA and Other Rare Diseases Study PPI group. They were invited to participate in this work through the Rare Dementia Support PPA Support Group (https://www.raredementiasupport.org/) The BCPPA and Other Rare Disease Study PPI group are responsible for providing guidance and advice on three work-packages being undertaken throughout the first author's current fellowship award. The lead author has, for example, recently been working with this group to explore the limitations of the PPA-COS Stage 2 study design and future dissemination plans for the work that have informed this protocol paper. The group meets four times annually and comprises five couples where one person in each couple has PPA. The group comprises three couples where one person has mild/moderate nfvPPA, two with mild/moderate lvPPA and their spousal partners, as well as one paid carer. Further individual PPI work is ongoing with people with svPPA and later stages of PPA who preferenced individual over group PPI contributions. All people with PPA and their partners involved in PPI activities are offered an honorarium and reimbursed for travel costs, in line with NIHR guidance [23]. Although PPI representatives reflect people with all major subtypes of PPA, there is limited socio-economic and ethnic diversity within the group, an issue the group plans to explore more broadly in the context of people with PPA accessing support services. PPI work will also be undertaken with clinical speech and language

For peer review only

therapists within the UK PPA speech and language therapist network, a group of clinical speech and language therapists with more than 80 members. Presentations and surveys will be used to consult speech and language therapists on their opinions about what the most important outcomes are for people with PPA.

2. *Collaborators*

Current collaborators, who are members of existing research networks such as the International PPA Speech and Language Therapy/Pathology Network and the Collaboration of Aphasia Triallists will be contacted regarding the study, and to identify new collaborators across World Health Organisation world regions and who speak and work with people with PPA in a variety of languages. Collaborators will also be identified through existing links with psychology, neuropsychology, occupational therapy and neurology disciplines, contacting authors from these disciplines who have published intervention studies and through larger networks such as the International Society on Frontotemporal Dementia (https://isftd.org) and the Include Network: For Global Equity in Language-Based Brain Health Research (https://www.gbhi.org/news-publications/include-network-global-equity-language-based-brain-health-research) .

3. *The study steering group*

In addition to the main research team (see author list), the study will be overseen by the BCPPA and other rare diseases steering group, comprising the lead author, AV, chaired by Dr Paul Conroy, attended by Dr Cath Mummery, Consultant Neurologist, Rosemary Townsend, Speech and Language Therapist in a third sector organisation, Hannah Luff, Speech and Language Therapist in an NHS trust, Phillip Robinson, carer of person with PPA and Samantha Stern, Neuropsychologist.

Study participants

Throughout this study, key stakeholders will be involved as participants. In line with previous work undertaken in the field of dementia research [14,15] three key stakeholder groups have been identified:

People living with PPA: People diagnosed with PPA of any variant.

Family care partners: Unpaid family care partner for a person living with PPA, may include spousal, partners, siblings, adult children, or close friends.

For peer review only

Researchers: Researchers from all backgrounds including speech and language, neurology, psychology and all those who identify as working current or recently, undertaking PPA-related research (i.e., as denoted by being a lead or co-author on dementia-related peer-reviewed publications or involvement in current dementia-related research).

Design

The COS-PPA will be developed over three stages: Stage 1) a systematic review of current measures described in the intervention research literature for PPA; Stage 2) consensus work to identify the most important outcomes for key stakeholders including people with PPA and their family members; and Stage 3) a modified Delphi consensus study with researchers working in the field of PPA intervention research to agree the core outcomes and measurement set. Importantly, the work will endeavour to reflect an international perspective to ensure it represents the needs of a diversity of key stakeholders and will thus be meaningful and relevant to the research and clinical needs of people with PPA across the world. Given some measurement tools may not be available in all languages, this study may provide a template for prioritising tool development in languages not covered by exisiting measures. This approach follows the COMET handbook [24], a guide for developing core sets of outcome and measurements. Figure 1 provides an overview of the workflow for the COS-PPA study.

For peer review

Figure 1: Workflow for COS-PPA

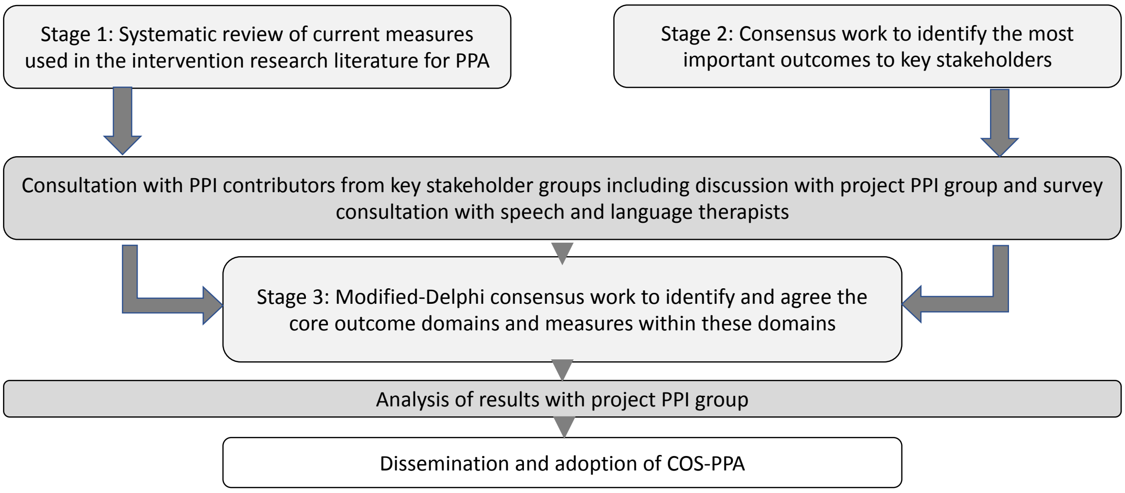

Stage 1: A systematic review of current measures used in the intervention research literature for PPA

The aim of this systematic review is to examine the speech, language and communication measures used to examine the effectiveness of interventions for PPA (behavioral, pharmacological or neuromodulation) to date in order to:

- Identify the constructs that are measured, and how they align with the WHO International Classification of Functioning, Disability and Health (ICF) domains.
- Identify relevance of these measures to the PPA variants; svPPA, lvPPA, nfvPPA and atypical PPA.
- Explore the psychometric properties of the measures using the COnsensus-based Standards for the selection of health Measurement INstruments (COSMIN) guidance [24] on reviewing outcome measures.

Procedures: This systematic review will replicate and update a recent review undertaken in the field of PPA interventions [25], employing the same search strategy but expanding this to include non-pharmacological interventions. Studies will be included that: a) describe original research (any design), b) are published in a peer-reviewed journal (exclusive of conference abstracts), c) investigate behavioral, pharmacological or neuromodulation treatment for speech and/or language, d) have been conducted with one or more persons with a diagnosis of PPA, and e) report treatment outcomes for at least one individual. Nine databases will be searched: Medline; CINAHL; (all via EBSCOhost); Embase; PsycInfo; ComDisDome; Scopus; and Web of Science, including non-English measures. All outcome measures extracted will be examined by the lead author AV and those that meet the above-mentioned inclusion criteria will be included in the study. Titles and abstracts of identified articles will be reviewed by the lead author and independently reviewed by a second author (CJDH) and assessed for inclusion or exclusion. All included articles will then undergo a second round of full text screening by AV and CJDH independently. Any discrepancies in ratings will be discussed until an agreement is reached by AV and CJDH.

Data extraction and analysis: All primary and secondary outcome measures of speech, language and communication reported in the final list of studies will be extracted and documented in a spreadsheet. Each measure will be considered in terms of the constructs it examines and how these align with the WHO ICF domains of impairment, activity, participation, environmental

For peer review only

factors (such as impact of health condition on communication partners) and communication related quality of life [27]. Data will be extracted from each article with regard to which PPA variant measures were used with (lvPPA, svPPA and nfvPPA). Finally, in line with the COSMIN guidance [26] on reviewing outcome measures, data will be sought and extracted on content validity, internal structure (i.e., structural validity and internal consistency, and/or Item Response Theory (IRT)/Rasch model fit); and where applicable the remaining measurement properties (i.e., reliability, measurement error, hypotheses testing, cross-cultural validity, criterion validity, and responsiveness). The full protocol for this review was registered on PROSPERO in December 2022: CRD42022367565.

Stage 2: Consensus work to identify the most important outcomes to key stakeholders

The important intervention outcomes for key stakeholders will be identified using group consensus methods. A Nominal Group Technique (NGT) protocol previously developed to meet the needs of people with stroke aphasia by co-author Dr Sarah Wallace [28], will be modified for people with PPA.

Recruitment: There is growing concern about the relevance of some COS to research in low- and middle-income countries, since participation in COS studies in those areas has been limited [12]. Therefore, to ensure representation from all WHO regions of the world, and ensure representation from low- and middle-income countries, researchers and research institutions have been approached to invite them to participate. Members of the CATs and the International PPA Speech and Language Therapy/Pathology network have agreed to participate as collaborators in the study. Collaborators across 14 countries have agreed to participate and have ethical approval including UK, Chile, Canada, Australia, India, Germany, Portugal, Italy, Israel, Spain, USA, Netherlands, Norway and Brazil. Having agreed to participate, a manual and slide deck, will be shared with collaborators instructing them on the methodology of how to run the groups (remotely where required by COVID-19 restrictions) in their respective countries. Collaborators have been asked to seek ethical approval or equivalent from their relevant institution and to translate materials where required.

Procedures: Collaborators will approach people with PPA, and their families via networks known to them in each country and invite them, via email, to participate in a one-off group meeting. Participants will complete consent procedures required by each collaborator's

For peer review only

institution. Collaborators will collate demographic data from participants on age, sex, type of PPA and time since diagnosis. Meetings will be held either online, via video conferencing, or in person, depending on the current COVID-19 restrictions and ethical approval guidance within the relevant country. Participants with PPA will attend one meeting and family members will attend a different meeting. Participants with PPA will be asked, "What would you most like to change about your communication and the way PPA affects your life?". Family members will be asked,"What would you most like to change about your family member's communication and the way PPA affects your life?". Following NGT [29,30] methods the participants will be invited to generate a list of items in response to the question, which will be shared in a round robin style with the remaining group members. Having collated a list of ideas, participants will be asked to identify their top three items in order of priority. A copy of the study manual is available in the supplementary material.

Analysis: Demographic data will be anonymised, and descriptive statistics comprising mean values will be calculated. The group facilitator (the collaborator in each country) will weight each answer (top items will be weighted with a 3, second with a 2 and third with a 1), and aggregate individual scores to produce a final list of results, identifying the top three rated items for each group (people with PPA and their families) in each country. Anonymised results collected from different countries will be shared with the lead author (AV), who will aggregate items in line with NGT methodology [29,30] and produce an overall list of results, and demographic data. Results will consider both an overall PPA outcomes list of results, and those that align with particular PPA variants.

Ethical approval: Ethical has been sought by each collaborator at their participating institutions (see xx) and participants will be consented based on local guidance.

Data collected from both Stage 1 & 2 will be presented to the project PPI group for analysis and discussion to agree the key constructs that should inform discussion at the final consensus group. Gaps in the data and reasons for this will be considered and will inform prioritisation of information for the final consensus meeting.

Stage 3: COS-PPA Consensus work to identify and agree core outcomes and measurement sets

Consensus work to agree a final COS-PPA with the team of international cross-disciplinary collaborators will be undertaken using Delphi consensus methods [29]. This will include

For peer review only

identifying and agreeing the core outcome measurement instruments for each of key constructs identified in Stages 1 and 2. Stage 3 work has been granted ethical approved by the Chairs of UCL Language and Cognition Department Ethics, Project ID LCD-2023-06.

Participants: Participants will be recruited via international networks of researchers working in the field of PPA intervention research. Given researchers in this field may include speech and language researchers, neuropsychology, psychology, neurology, occupational therapy and other groups it will be essential to explore networks within and outside of the International PPA Speech and language Therapy / Pathology network and CATS. Researchers and authors of studies identified in the Stage 1 systematic review will be contacted, in addition to contacting researchers via additional networks including the International Society of Fronto-Temporal Dementia (ISFTD). The authors will purposefully aim to recruit representatives of researchers across a range of countries, without overly biasing representation from one country.

Procedures: Emails will be sent inviting researchers to participate in the study, should they agree by return of email, they will be sent a link to complete an online consent form and brief survey collecting demographic information (including affiliation, professional background, research interests, country of current work, qualification, number of PPA participants seen as part of research, languages in which research undertaken) and availability to attend a meeting hosted on a video conferencing platform.

Prior to attending video conferencing meetings, participants will be asked to complete an online vote rating the importance of outcome constructs (selected based on data collated in Stage 2 of the study). Participants will rate each construct on a scale of importance with 9 being the most important and 1 being the least important. Rankings of 7-9 indicate critical importance, 4-6 outcomes that are important but not critical, while ratings of 1-3 are of limited importance using the Grading of Recommendations Assessment, Development and Evaluations (GRADE) scale [31]. Participants will be invited to put other constructs forward that they think are important but had not been included in the survey. They will also be asked about which constructs require different measurements for individual PPA variants and which do not. Results of the first round of voting will be re-disseminated and participants will be asked to re-vote on the importance of these constructs.

Only participants who have completed the online ratings will be invited to participate in online meetings. At least two online meetings, hosted on Zoom, will be held to capture participants

For peer review only

across different zones. Meetings will be facilitated by an independent facilitator, not affiliated with research in this field. For each construct participants will be presented with measures identified in the Stage 1 systematic review. Participants will be provided with a description of each measure, including its psychometric properties and languages in which it is available. Participants will then be asked to vote for relevant measures within each construct domain. Following the group meetings, results across groups will be aggregated. Participants will be invited to re-vote on measures via email.

Analysis: Demographic information will be analysed using descriptive statistics. Anonymised voting data collected in the modified Delphi consensus study will be aggregated and data italicised for presentation.

Dissemination:

Work undertaken at Stages 1, 2 and 3 will be published in open-access peer reviewed journal articles and presented at international scientific conferences. The results will additionally be published in clinical practice magazines in the UK, the Royal College of Speech and Language Therapy Bulletin magazine and Practical Neurology, and equivalent non-UK practice outlets identified by collaborators in relevant participating countries. The work will also be submitted to the World Health Organization's Global Observatory Knowledge Exchange Platform [32], a platform designed specifically to share knowledge about dementia research in response to the Global Action Plan on the Public Health Response to Dementia 2017-2025 [33].

Prior to publication, findings from the Stage 2 work to identify the most important outcomes for key stakeholders will be shared with participating international collaborators in the form of a draft manuscript. They will be invited to be co-authors, and to comment on the manuscript prior to submission to a peer reviewed journal. Additionally, it will be suggested that they share co-produced accessible with local participants.

Consensus work undertaken at Stage 3 will be shared by email with participants. Participants will be invited, at the outset, to collaborate on a peer reviewed manuscript, as co-authors. Having remained involved in the consensus process, participants will be reminded of this and invited to collaborate and comment on a draft manuscript. As co-authors on the paper, they will assist in the dissemination of the results as well as receiving continuing updates on the research study.

For peer review only

The PPI contributors and the study steering group will be acknowledged in all submitted manuscripts and scientific conference presentations.

Discussion:

This protocol describes the development of a COS for PPA (COS-PPA) following three stages to identify the current measures used in the intervention research literature, what is important to key stakeholders internationally and finally a modified-Delphi consensus exercise to agree the COS. There is currently no published COS for PPA, and existing COSs for Dementia [14,15] and non-progressive aphasia [16,17] do not address the unique and specific needs of this group. The current research literature on PPA interventions use a variety of outcomes [8], limiting comparability across studies and moreover do not address what is important to key stakeholders. Identifying measures that reflect the needs of people with all syndroms PPA variants will be challenging, It is essential that we understand the priorities of this seldom-heard group of people with speech, language, and communication difficulties and that we ensure we embed these in our research design. Using a COS could allow for combination of datasets, which, in the current setting of smaller studies would increase the power of the intervention studies and increase our understanding of efficacy of interventions for PPA. Given our collaborations internationally, the intention is for this COS to be used internationally, thus the dissemination plan includes both publications in peer reviewed journals and international dissemination platforms. We anticipate that there will be few measures available that have been translated into different languages. In fact, it is likely that there may be no suitable measures to address all the identified constructs and that are validated or developed for use with people with PPA. This highlights the potential for this research to inform and help prioritise a program of work to develop new measures and support the translation and validation of measures for people with PPA across different languages. This will potentially have far-reaching implications beyond the scope of PPA, extending to people living with other dementia types, who also have speech, language, and communication needs such as Posterior Cortical Atrophy [34], Frontotemporal Dementia, Young Onset Alzheimer's Disease, Typical Alzheimer's Disease and Lewy Body Dementia [35].

Abbreviations:

Trial Status:

For peer review only

Stage 1 Systematic review searches March 2023, data extraction May 2023

Stage 2 Nominal Groups with people with PPA July 2021-May 2023, Survey of Speech and Language Therapists June 2023

Stage 3 Modified Delphi Consensus methods July 2023-October 2023

Ethical approval:

All aspects of the study will be conducted according to the Declaration of Helsinki.

The Stage 2 UK collaboration is being undertaken as part of the Rare Dementia Support (RDS) Impact Study which received approval from the UCL Research Ethics Committee (8545/004: Rare Dementia Support Impact Study). All consent sessions will be video recorded, in line with the approved procedure outlined in the RDS Impact study protocol (Brotherhood et al., 2020). For the collaboration with Dr Carolina Mendez in Chile, ethical approval has been granted by the Pontificia Universidad Catolixa de Chile Ethics Committee, ID no. 190510002. For the collaboration with Dr Regina Jokel in Canada, ethical approval has been granted by Baycrest, Research Ethics Board REB 22-37. For the collaboration with Dr Jade Cartwright and Dr Cathy Taylor-Rubin in Australia ethical approval has been granted by Southeastern Sydney Local Health District HREC 2022/ETH02740. For the collaboration with Dr Avanthi Paplikar in India ethical approval has been granted by the Bangalore Speech and Hearing Research Foundation. For the collaboration with Prof Marcus Meinzer, Anna Rysop and Nina Unger in Germany ethical approval has been granted by Griefswald University Ethics Committee, Germany, Reference BB 130/22. For the collaboration with De Ines Cadorio in Portugal ethical approval has been granted by Ethics Committee of the University Fernando Pessoa Prot n. 50/C.E> del 28/02/22. For the collaboration with Dr Petronilla Battista in Italy, ethical approval was granted by the ethics Committee of the IRCCS Giovanni Paolo II Bari, Prot. n. 80/CE Maugeri on 17/02/2022. For the collaboration with Dr Adi Lifshitz Ben basat and Hagit Bar-Zeev in Israel ethical approval was granted by the Ariel University Ethics Board, Israel. For the collaboration with Dr Maya Henry and Carly Milanski in America ethical approval was given by the Office of Research Support and Compliance and the University of Texas at Austin's Institutional Review Board IRB ID STUDY00000717-MOD06. For the collaboration with Dr Sandra Weilart, Dr Lize Jiskoot, Janna Vanegmond, Heleen Hendriksen and Antoinette Keulen in the Netherlands ethical approval was granted by Amsterdam UMC under the number 2023.0098. For the collaboration with Dr Monica Norvik in Norway ethical approval was

For peer review only

granted by the Norwegian Agency for Shared Services in Education and Research (SIKT) ref: 865145. For collaboration with Maria Isabel d'Avila Freitas in Brazil ethical approval was granted by Hospital of Clinics- Faculty of medicine – Uni of Sao Paulo (USP) ref: 4.142.664.

Stage 3 work has been granted ethical approved by the Chairs of UCL Language and Cognition Department Ethics, Project ID LCD-2023-06. All participants involved will be asked to complete a written form consent before participation in the Delphi survey.

Funding sources:

This study was initially funded by a UCL Grand Challenge awarded to AV and CJH in 2020, and consequently by an NIHR Advanced Fellowship NIHR302240 to AV. Sarah J. Wallace is funded by a National Health and Medical Research Council (NHMRC) Investigator Grant (1175821).

Conflicts of interest:

All authors declare that there are no known conflicts of interest.

Author contributions:

AV is the chief investigator, she conceived the study, led the proposal and protocol development.

CJDH contributed to the study design and development of the proposal.

SW and DC contributed to the study design.

RV, MH and JDW contributed to finalising the proposal.

All authors have contributed to the manuscript preparation.

Acknowledgements:

The authors would like to acknowledge the invaluable assistance and advice of all the PPI contributors, the study steering group (Dr Paul Conroy, Dr Cath Mummery, Rosemary Townsend, Hannah Luff, Phillip Robinson and Samantha Stern). We would also like to thank all collaborators across all collaborating countries; Dr Carolina Mendez in Chile, Dr Regina Jokel in Canada, Dr Jade Cartwright and Dr Cathy Taylor-Rubin in Australia, Dr Avanthi Paplikar in India, Prof Marcus Meinzer, Anna Rysop and Nina Unger in, Dr Ines Cadorio in Portugal, Dr Petronilla Battista in Italy, Dr Adi Lifshitz Ben basat and Hagit Bar-Zeev in Israel, Dr Maya Henry and Carly Milanski in America, Dr Sandra Weilart, Dr Lize Jiskoot, Janna

For peer review only

Vanegmond, Heleen Hendriksen and Antoinette Keulen in the Netherlands, Dr Monica Norvik in Norway, Maria Isabel d'Avila Freitas in Brazil, Dr Ibrahim Can Yasa in Turkey and Antoine Renard in France.

References:

1. Gorno-Tempini ML, Hillis AE, Weintraub S, Kertesz A, Mendez M, Cappa SF, Ogar JM, Rohrer JD, Black S, Boeve BF, Manes F. Classification of primary progressive aphasia and its variants. Neurology. 2011 Mar 15;76(11):1006-14.
2. Marshall CR, Hardy CJ, Volkmer A, Russell LL, Bond RL, Fletcher PD, Clark CN, Mummery CJ, Schott JM, Rossor MN, Fox NC. Primary progressive aphasia: a clinical approach. Journal of neurology. 2018 Jun;265:1474-90.
3. Ruksenaite J, Volkmer A, Jiang J, Johnson JC, Marshall CR, Warren JD, Hardy CJ. Primary progressive aphasia: toward a pathophysiological synthesis. Current neurology and neuroscience reports. 2021 Mar;21:1-2.
4. Carthery-Goulart MT, Silveira AD, Machado TH, Mansur LL, Parente MA, Senaha ML, Brucki SM, Nitrini R. Nonpharmacological interventions for cognitive impairments following primary progressive aphasia: A systematic review of the literature. Dementia & Neuropsychologia. 2013 Jan;7:122-31.
5. Cadório I, Lousada M, Martins P, Figueiredo D. Generalization and maintenance of treatment gains in primary progressive aphasia (PPA): A systematic review. International journal of language & communication disorders. 2017 Sep;52(5):543-60
6. Cotelli M, Manenti R, Ferrari C, Gobbi E, Macis A, Cappa SF. Effectiveness of language training and non-invasive brain stimulation on oral and written naming performance in Primary Progressive Aphasia: A meta-analysis and systematic review. Neuroscience & Biobehavioral Reviews. 2020 Jan 1;108:498-525.
7. Nissim NR, Moberg PJ, Hamilton RH. Efficacy of noninvasive brain stimulation (tDCS or TMS) paired with language therapy in the treatment of primary progressive aphasia: an exploratory meta-analysis. Brain Sciences. 2020 Aug 28;10(9):597.
8. Volkmer A, Spector A, Meitanis V, Warren JD, Beeke S. Effects of functional communication interventions for people with primary progressive aphasia and their caregivers: A systematic review. Aging & mental health. 2020 Sep 1;24(9):1381-93.
9. Volkmer A, Walton H, Swinburn K, Spector A, Warren JD, Beeke S. Results from a randomised controlled pilot study of the Better Conversations with Primary Progressive

Aphasia (BCPPA) communication partner training program for people with PPA and their communication partners.

10. Williamson PR, Altman DG, Bagley H, Barnes KL, Blazeby JM, Brookes ST, Clarke M, Gargon E, Gorst S, Harman N, Kirkham JJ. The COMET handbook: version 1.0. Trials. 2017 Jun;18:1-50.
11. Williamson PR, Altman DG, Blazeby JM, Clarke M, Devane D, Gargon E, Tugwell P. Developing core outcome sets for clinical trials: issues to consider. Trials. 2012 Dec;13(1):1-8.
12. Kirkham JJ, Davis K, Altman DG, Blazeby JM, Clarke M, Tunis S, Williamson PR. Core outcome Set-STAndards for development: the COS-STAD recommendations. PLoS medicine. 2017 Nov 16;14(11):e1002447.
13. Harding, A. J., Morbey, H., Ahmed, F., Opdebeeck, C., Wang, Y. Y., Williamson, P., ... & Reilly, S. (2018). Developing a core outcome set for people living with dementia at home in their neighbourhoods and communities: study protocol for use in the evaluation of non-pharmacological community-based health and social care interventions. Trials, 19, 1-13.
14. Harding, A. J., Morbey, H., Ahmed, F., Opdebeeck, C., Lasrado, R., Williamson, P. R., ... & Reilly, S. T. (2019). What is important to people living with dementia?: the 'long-list'of outcome items in the development of a core outcome set for use in the evaluation of non-pharmacological community-based health and social care interventions. BMC geriatrics, 19(1), 1-12.
15. Reilly, S. T., Harding, A. J., Morbey, H., Ahmed, F., Williamson, P. R., Swarbrick, C., ... & Keady, J. (2020). What is important to people with dementia living at home? A set of core outcome items for use in the evaluation of non-pharmacological community-based health and social care interventions. Age and ageing, 49(4), 664-671.
16. Wallace, S. J., Worrall, L., Rose, T., Le Dorze, G., Breitenstein, C., Hilari, K., ... & Webster, J. (2019). A core outcome set for aphasia treatment research: The ROMA consensus statement. International journal of stroke, 14(2), 180-185.
17. Wallace, S. J., Worrall, L., Rose, T. A., Alyahya, R. S., Babbitt, E., Beeke, S., ... & Dorze, G. L. (2022). Measuring communication as a core outcome in aphasia trials: Results of the ROMA-2 international core outcome set development meeting. International Journal of Language & Communication Disorders.
18. Loizidou M, Brotherhood E, Harding E, Crutch S, Warren JD, Hardy CJ, Volkmer A. 'Like going into a chocolate shop, blindfolded': What do people with primary progressive aphasia

want from speech and language therapy?. International Journal of Language & Communication Disorders. 2022 Nov 30.

19. Hardy, C. J., Taylor-Rubin, C., Taylor, B., Harding, E., Suarez Gonzalez, A., Jiang, J., ... & Warren, J. D. (2023). Symptom-led staging for primary progressive aphasia. *medRxiv*, 2023-03.
20. World Health Organisation's regions of the world https://www.who.int/about/who-we-are/regional-offices
21. Kirkham JJ, Gorst S, Altman DG, Blazeby JM, Clarke M, Tunis S, Williamson PR, COS-STAP Group Adrian Aldcroft Heather Bagley Bronwen Connolly Sarah Gorst Jamie J. Kirkham Tianjing Li Karen Matvienko-Sikar Victoria Thomas Sean Tunis Paula R. Williamson. Core outcome Set-STAndardised protocol items: the COS-STAP statement. Trials. 2019 Dec;20:1-7.
22. Volkmer A, Broomfield K, editors. Seldom Heard Voices in Service User Involvement: The how and why of meaningful collaboration. J & R Press Limited; 2022.
23. NIHR Patient and Public Involvement Resources (Viewed March 2023) https://www.nihr.ac.uk/documents/ppi-patient-and-public-involvement-resources-for-applicants-to-nihr-research-programmes/23437
24. Williamson, P.R., Altman, D.G., Bagley, H. *et al.* The COMET Handbook: version 1.0. *Trials* **18** (Suppl 3), 280 (2017). https://doi.org/10.1186/s13063-017-1978-4
25. Wauters et al. Behavioral treatment for speech and language in primary progressive aphasia and primary progressive apraxia of speech: A systematic review (Under Review)
26. COSMIN guidance (Viewed March 2023) https://static-content.springer.com/esm/art%3A10.1186%2Fs13063-016-1555-2/MediaObjects/13063_2016_1555_MOESM2_ESM.pdf
27. WHO International Classification of Functioning, Disability and Health (ICF) domains
28. Wallace SJ, Worrall L, Rose T, Le Dorze G, Cruice M, Isaksen J, Kong AP, Simmons-Mackie N, Scarinci N, Gauvreau CA. Which outcomes are most important to people with aphasia and their families? An international nominal group technique study framed within the ICF. Disability and rehabilitation. 2017 Jul 3;39(14):1364-79.
29. McMillan SS, King M, Tully MP. How to use the nominal group and Delphi techniques. International journal of clinical pharmacy. 2016 Jun;38:655-62.
30. McMillan SS, Kelly F, Sav A, Kendall E, King MA, Whitty JA, Wheeler AJ. Using the nominal group technique: how to analyse across multiple groups. Health Services and Outcomes Research Methodology. 2014 Sep;14:92-108.

For peer review only

31. Guyatt GH, Oxman AD, Vist GE, Kunz R, Falck-Ytter Y, Alonso-Coello P, et al. GRADE: an emerging consensus on rating quality of evidence and strength of recommendations. Bmj. 2008;336(7650):924-6.
32. World Health Organization's Global Observatory Knowledge Exchange Platform (Viewed March 2023) https://www.who.int/data/gho/data/themes/global-dementia-observatory-gdo
33. Global Action Plan on the Public Health Response to Dementia 2017-2025. (Viewed March 2023) https://www.who.int/publications/i/item/global-action-plan-on-the-public-health-response-to-dementia-2017---2025
34. Volkmer A, Farrington-Douglas C, Crutch S, Beeke S, Warren J, Yong K. Better conversations: a language and communication intervention for aphasia in posterior cortical atrophy. Neurocase. 2022 Jul 4;28(4):356-63.
35. Suárez-González A, Cassani A, Gopalan R, Stott J, Savage S. When it is not primary progressive aphasia: a scoping review of spoken language impairment in other neurodegenerative dementias. Alzheimer's & Dementia: Translational Research & Clinical Interventions. 2021;7(1):e12205.

For peer review only

BMJ Open

COS- PPA: Protocol to develop a core outcome set for Primary Progressive Aphasia

Journal:	*BMJ Open*
Manuscript ID	bmjopen-2023-078714.R1
Article Type:	Protocol
Date Submitted by the Author:	06-Mar-2024
Complete List of Authors:	Volkmer, Anna; University College London, Department of Psychology and Language Sciences<br>Copland, David; The University of Queensland - Saint Lucia Campus, School of Health and Rehabilitation Sciences; The University of Queensland, Queensland Aphasia Research Centre<br>Henry, Maya L.; The University of Texas at Austin<br>Warren, Jason; UCL,<br>Varley, Rosemary; University College London, Department of Psychology and Language Sciences<br>Wallace, Sarah J.; The University of Queensland, School of health and rehabilitation sciences; The University of Queensland<br>Hardy, Chris ; University College London, Dementia Research Centre
**Primary Subject Heading**:	Neurology
Secondary Subject Heading:	Rehabilitation medicine, Patient-centred medicine, Communication, Evidence based practice
Keywords:	Dementia, Speech pathology < OTOLARYNGOLOGY, Patient Reported Outcome Measures

SCHOLARONE™
Manuscripts

Title:

COS- PPA: Protocol to develop a core outcome set for Primary Progressive Aphasia

Volkmer, Anna.[1] (a.volkmer.15@ucl.ac.uk), Copland, David.[3,4], Henry, Maya L.[6], Warren, Jason D.[2], Varley, Rosemary[1]., Wallace, Sarah J.[3,4] Hardy, Chris J.D.[2]

Affiliations:

1. Department of Psychology and Language Sciences, University College London, London, UK.
2. Dementia Research Centre, Department of Neurodegenerative Disease, UCL Queen Square Institute of, Neurology, University College London, London, UK
3. Queensland Aphasia Research Centre, School of Health and Rehabilitation Sciences, The University of Queensland, Brisbane, Australia
4. Surgical Treatment and Rehabilitation Service (STARS) Education and Research Alliance, The University of Queensland and Metro North Health, Queensland, Australia
5. Departments of Speech, Language, and Hearing Sciences and Neurology, University of Texas, Austin, USA.

Contact information:

a.volkmer.15@ucl.ac.uk

University College London

Chandler House

2 Wakefield Street

London

WC1N 1PF

For peer review only

Abstract:

Introduction:

The term Primary Progressive Aphasia (PPA) describes a group of language-led dementias. Disease-modifying treatments that delay, slow or reverse progression of PPA are currently lacking, though a number of interventions to manage the symptoms of PPA, have been developed in recent years. Unfortunately, studies exploring the effectiveness of these interventions have used a variety of different outcome measures, limiting comparability. Typically, these measures do not necessarily assess what is important to key stakeholders. Existing core outcome sets (COS) for dementia and non-progressive aphasia do not meet the needs of people with primary progressive aphasia, highlighting a need to develop a specific core outcome set for PPA.

Methods and analysis: This protocol describes a three-stage study to identify a COS for Primary Progressive Aphasia interventions in research and clinical practice. The Stage 1 systematic review will identify existing speech, language and communication measures used to examine the effectiveness of interventions for PPA in the research literature. Employing a Nominal Group Technique, Stage 2 will identify the most important outcomes to people with PPA and their families. The data collected in Stages 1 and 2 will be jointly analysed with the project PPI group and will inform the Stage 3 modified Delphi consensus study to identify a core outcome measurement set for PPA amongst a range of research disciplines undertaking intervention studies for people with PPA.

Ethics and dissemination: Ethical approval for Stage 2 of the study has been sought individually in each country at collaborating institutions and is stated in detail in the manuscript. Stage 3 has been granted ethical approved by the Chairs of UCL Language and Cognition Department Ethics, Project ID LCD-2023-06. Work undertaken at Stages 1, 2 and 3 will be published in open-access peer reviewed journal articles and presented at international scientific conferences.

Trial registration: Registered with COMET March 2021: https://www.comet-initiative.org/Studies/Details/1871

Strengths and Limitations:

For peer review only

- This protocol describes a rigorous three stage study, incorporating the current literatures, views of people with PPA, their family members and international, interdisciplinary researchers to identify a Core Outcome Set for Primary Progressive Aphasia.
- Collaborating research sites have been identified across 15 different countries worldwide.
- Translation of consensus materials from and to English will be undertaken by the research collaborators.
- Consensus groups will be facilitated by speech and language researchers.
- The Patient and Public Involvement advisory group will support the analysis of results.

Keywords:

Primary Progressive Aphasia

Core Outcome Set

Speech and Language Therapy

Interventions

Background:

Primary Progressive Aphasia (PPA) is a term used to describe a group of speech and language-led dementias that advance inexorably over time [1,2,3]. Three major PPA syndromes have been identified. Semantic variant PPA (svPPA) presents with difficulties in producing and understanding words and tends to be associated with frontotemporal lobar degeneration. Nonfluent variant (nfvPPA) is characterised by a motor-speech disorder called speech apraxia, which presents as groping effortful speech, and/or difficulties in using grammar (agrammatism) and also tends to be associated with frontotemporal lobar degeneration. PPA Apraxia of Speech (PPA AOS), describes a 'purer' form of nfvPPA, and can be further fractioned into phonetic or prosodic subtypes, but is generally considered part of the broader nfvPPA syndrome . Finally, Logopenic variant PPA (lvPPA) results in difficulties in word retrieval and phonological working memory and tends to be associated with Alzheimer's pathology [3].

There is currently no disease-modifying treatment for any form of PPA and symptomatic pharmacological therapies have also not shown evidence of effectiveness across all PPA variants. Speech and language therapists, psychologists and neuroscientists across the world have however, developed multiple tailored interventions for people with PPA [4,5,6,7,8]. Yet, research examining the effectiveness of these interventions has generally focused on case studies, and cohorts with small sample sizes. Only one randomised controlled trial has been reported in the intervention literature to date [9]. Importantly, there has also been huge variability in outcome measures used across all these studies [4,8].

Given the rarity and clinical heterogeneity of this disease, being able to group data would strengthen outcomes and knowledge about generalisability of interventions for people with PPA. Similarly, comparing data can enhance evidence-based clinical decision making and service development [10]. Ensuring that measures are meaningful to key stakeholders, including people with the disease, their families and relevant health care professionals will be vital to this process [11]. The huge variability in outcomes used within the PPA intervention literature places limitations on aggregation of outcome data, comparisons across studies and ultimately the delivery of appropriate clinical services.

A Core Outcome Set (COS) is an agreed set of outcomes that are measured and reported in intervention studies related to a particular health condition [11]. The main reason to develop a COS is to allow for comparison across similar trials, standardise reporting to reduce selective and/or biased reporting, and make results more relevant [12]. There has been previous work undertaken to develop a COS for use in the evaluation of non-pharmacological interventions for all-cause dementia which identified a long list of 54 items [13,14]. Though this generic list included communication, the list was not salient to interventions with the similar objectives (it encompassed a wide variety) and did not reflect the views of stakeholders internationally [13,14]. The authors of the dementia COS also acknowledged that the constructs identified in this work are broad and overlapping and therefore lacking specificity [15] in terms of the measures to be used.

Work has been undertaken to identify a COS for post-stroke aphasia. The Research Outcome Measurement in Aphasia (ROMA)-COS identified five essential outcome constructs and appropriate measurement instruments that address each domain [16,17] including: language, communication, patient-reported satisfaction with treatment and impact of treatment,

For peer review only

emotional wellbeing, and quality of life. Importantly, the scope of the ROMA-COS focused on rehabilitation of non-progressive aphasia. Given people with PPA are living with a progressive disease, intervention outcomes will be different to those living with an acute onset, and potentially improving aphasia [18]. Additionally, given the heterogeneity of PPA, a COS should include consideration of the value of different outcomes for different PPA syndromes [19]. We have no roadmap at present for determining or evaluating intervention outcomes in PPA and it presents radically different challenges to stroke aphasia (the current standard for aphasia interventions) - both due to its intrinsically progressive nature but also because it entails significant issues with nonverbal cognition and behaviour over the course of the illness that interact with communication function- thus there is a fundamental need to reorient researchers and clinicals to PPA [18, 19, 20]. In summary, there is a need for a specific COS, that details key measures addressing the needs of people with PPA.

There is also an urgent need to improve access to care and supports in PPA for people from socioeconomic, linguistic and culturally diverse backgrounds. It is anticipated that if constructs identified are in anyway similar to the ROMA-COS, they will include constructs relating to participation, capabilities and wellbeing that traverse linguistic contexts. Therefore. including people with PPA and their families from a diverse range of linguistic and cultural backgrounds in the development of the COS is essential to capture the voices of people from these underserved communities. To address potential barriers to implementation of measures, incorporating an international and cross-disciplinary stakeholder perspective will promote uptake of the COS across the research community internationally. This, in turn, will promote opportunities for future international collaborations.

The aim of this international cross-disciplinary collaboration is to develop a core set of outcome measures for researchers in the field of PPA interventions. Identifying 'what' and 'how' best to measure outcomes will inform future developments in PPA intervention research, as well as improving the impact of the work being undertaken. This, in turn, will benefit individuals with PPA by increasing access to evidence-based interventions. Thus, the objectives of this study are:

- To extract the speech, language and communication measures used to examine the effectiveness of interventions for PPA to date in the research literature.

- To identify the most important outcomes to key stakeholders, including people with PPA, their families and speech and language therapists.
- To identify outcomes that address the needs of people with different PPA variants.
- To achieve consensus on a COS for PPA (the PPA-COS) amongst a range of research disciplines undertaking intervention studies for people with PPA.

Scope:

1. The health condition and population covered by this COS:
   The PPA-COS covers people with PPA (inclusive of PPAOS), as defined by the current internationally accepted diagnostic criteria [1].

2. The interventions covered by this COS:
   The PPA-COS covers intervention research that aims to affect all domains of speech, language and communication, including communication related quality of life and wellbeing, for people living with PPA and their communication partners.

3. The settings covered by this COS:
   The setting covered by this COS-PPA covers intervention research and clinical delivery of interventions to people with PPA internationally, with the goal of capturing key stakeholders across all major World Health Organisation's regions of the world [21] who speak and work with people with PPA in a variety of languages. We anticipate that there may be issues with measures that have not yet been translated into languages other than English and will consider the implications of this during this process.

Methods:

The 11 minimum standards outlined in the COS-Standards for Development Recommendations (COS-STAD) [12] informed the development of this protocol to ensure the scope, stakeholders and consensus processes were all addressed in line with these recommendations. The COS-Standardised Protocol (COS-STAP) [22] Protocol Items consisting of a checklist of 13 items and accompanying COS-STAP Explanation and Elaboration (E+E) [22] document were also consulted to ensure the COS-PPA development plans, as well as stakeholder involvement and consensus processes, were adequately addressed (see supplementary information for completed COS-STAP reporting checklist). The COS-PPA was registered on the COMET website in

For peer review only

March 2021: https://www.comet-initiative.org/Studies/Details/1871. In addition, the Stage 1 systematic review was preregistered on PROSPERO in December 2022: CRD42022367565.

Stakeholders:

Members of the research team

1. *Patient and Public Involvement (PPI)*

   In line with guidance from the National Institute for Health and Care Research (NIHR) PPI work encourages researchers to do research with people 'with' rather than 'to' people with lived experience [23]. The development of this study proposal was informed by PPI work undertaken during the Better Conversations with PPA (BCPPA) randomised controlled pilot study [9]. PPI collaborators identified that measurement tools needed to reflect what was important to people living with PPA [24]. The current COS-PPA study is overseen by the BCPPA and Other Rare Diseases Study PPI group. Members of this PPI group were invited from the Rare Dementia Support PPA Support Group (https://www.raredementiasupport.org/) using purposeful recruitment strategies. The BCPPA and Other Rare Disease Study PPI group are responsible for providing guidance and advice on three work-packages being undertaken throughout the first author's current NIHR funded fellowship award. The lead author has, for example, recently been working with this group to explore the limitations of the PPA-COS Stage 2 study design and future dissemination plans for the work that has informed this protocol paper. The group meets four times annually and comprises five couples where one person in each couple has PPA. The group comprises three couples where one person has mild/moderate nfvPPA, two with mild/moderate lvPPA and their spousal partners, as well as one paid carer. Further individual PPI work is ongoing with people with svPPA and those in later stages of PPA who preferred individual over group PPI contributions. All people with PPA and their partners involved in PPI activities are offered an honorarium and reimbursed for travel costs, in line with NIHR guidance [23]. Although PPI representatives reflect people with all major subtypes of PPA, there is limited socio-economic and ethnic diversity within the group, an issue the group plans to explore more broadly in the context of people with PPA accessing support services. PPI work will also be undertaken with clinical speech and language therapists within the UK PPA speech and language therapist network: a group of clinical speech and language therapists with more than 80 members. Presentations and surveys will be

For peer review only

used to consult speech and language therapists for their opinions on the most important outcomes for people with PPA.

2. *Collaborators for Stages 2 & 3 of the study*

   Current collaborators, who are members of existing research networks such as the International PPA Speech and Language Therapy/Pathology Network and the Collaboration of Aphasia Trialists will be contacted regarding the study, and new collaborators across World Health Organisation world regions and who speak and work with people with PPA in a variety of languages will be identified. These networks will be asked, via their executive committees and leads to disseminate information about the study to all relevant working groups. Collaborators will also be identified through existing links with psychology, neuropsychology, occupational therapy and neurology disciplines, contacting authors from these disciplines who have published intervention studies and through larger networks such as the International Society on Frontotemporal Dementia (https://isftd.org) and the Include Network: For Global Equity in Language-Based Brain Health Research (https://www.gbhi.org/news-publications/include-network-global-equity-language-based-brain-health-research) .

3. *The study steering group*

   In addition to the main research team (see author list), the study will be overseen by the BCPPA and other rare diseases steering group, comprising the lead author, AV, chaired by Dr Paul Conroy, attended by Dr Cath Mummery, Consultant Neurologist, Rosemary Townsend, Speech and Language Therapist in a third sector organisation, Hannah Luff, Speech and Language Therapist in an NHS trust, Phillip Robinson, carer of person with PPA and Samantha Stern, Neuropsychologist.

Study participants

Throughout this study, key stakeholders will be involved as participants. In line with previous work undertaken in the field of dementia research [14,15] three key stakeholder groups have been identified:

People living with PPA: People diagnosed with PPA of any variant.

Family care partners: Unpaid family care partner for a person living with PPA, may include spousal, partners, siblings, adult children, or close friends.

Researchers: Researchers from all backgrounds including speech and language, neurology, psychology and all those who identify as working currently or recently, undertaking PPA-related research (i.e., as denoted by being a lead or co-author on dementia-related peer-reviewed publications or involvement in current dementia-related research).

Design

The COS-PPA will be developed over three stages: Stage 1) a systematic review of current measures described in the intervention research literature for PPA; Stage 2) consensus work to identify the most important outcomes for people with PPA and their family members; and Stage 3) a modified Delphi consensus study with researchers working in the field of PPA intervention research to agree the core outcomes and measurement set.

Importantly, the work will endeavour to reflect an international perspective to ensure it is representative of the needs of people with PPA and their families across the world. Given some measurement tools may not be available in all languages, this study may provide a template for prioritising tool development in languages not covered by existing measures.

This approach follows the COMET handbook [25], a guide for developing core sets of outcome and measurements. Figure 1 provides an overview of the workflow for the COS-PPA study.

Figure 1: Workflow for COS-PPA

Stage 1: A systematic review of current measures used in the intervention research literature for PPA

The aim of this systematic review is to examine the speech, language and communication measures used to examine the effectiveness of interventions for PPA (behavioral, pharmacological or neuromodulation) to date in order to:

- Identify the constructs that are measured, and how they align with the WHO International Classification of Functioning, Disability and Health (ICF) domains.

For peer review only

- Identify relevance of these measures to the PPA variants; svPPA, lvPPA, nfvPPA and atypical PPA.
- Explore the psychometric properties of the measures using the COnsensus-based Standards for the selection of health Measurement INstruments (COSMIN) guidance [24] on reviewing outcome measures.

Procedures: This systematic review will replicate and update a recent review undertaken in the field of PPA interventions [26], employing the same search strategy but expanding this to include non-pharmacological interventions. Studies will be included that: a) describe original research (any design), b) are published in a peer-reviewed journal (exclusive of conference abstracts), c) investigate behavioral, pharmacological or neuromodulation treatment for speech and/or language, d) have been conducted with one or more persons with a diagnosis of PPA, and e) report treatment outcomes for at least one individual. Nine databases will be searched: Medline; CINAHL; (all via EBSCOhost); Embase; PsycInfo; ComDisDome; Scopus; and Web of Science. Papers/measures published in languages other than English will not be excluded from the study. All outcome measures extracted will be examined by the lead author AV and those that meet the above-mentioned inclusion criteria will be included in the study. Titles and abstracts of identified articles will be reviewed by the lead author and independently reviewed by a second author (CJDH) and assessed for inclusion or exclusion. All included articles will then undergo a second round of full text screening by AV and CJDH independently. Any discrepancies in ratings will be discussed until an agreement is reached by AV and CJDH.

Data extraction and analysis: All primary and secondary outcome measures of speech, language and communication reported in the final list of studies will be extracted and documented in a spreadsheet. Each measure will be considered in terms of the constructs it examines and how these align with the WHO ICF domains of impairment, activity, participation, environmental factors (such as impact of health condition on communication partners) and communication related quality of life [27]. Data will be extracted from each article with regard to which PPA variant measures were used with (lvPPA, svPPA, nfvPPA or no specific PPA variant, taking into account current [1] and previous PPA classification criteria [28,29]). Finally, in line with the COSMIN guidance [30] on reviewing outcome measures, data will be sought and extracted on content validity, internal structure (i.e., structural validity and internal consistency, and/or Item Response Theory (IRT)/Rasch model fit); and where applicable the remaining measurement properties (i.e., reliability, measurement error, hypotheses testing, cross-cultural

For peer review only

validity, criterion validity, and responsiveness). The full protocol for this review was registered on PROSPERO in December 2022: CRD42022367565.

Stage 2: Consensus work to identify the most important outcomes to people with PPA and their families

The important intervention outcomes for people with PPA and their families will be identified using group consensus methods. A Nominal Group Technique (NGT) protocol previously developed to meet the needs of people with stroke aphasia by co-author Dr Sarah Wallace [31], will be modified for people with PPA.

Collaborating research sites: There is growing concern about the relevance of some COS to research in low- and middle-income countries, since participation in COS studies in those areas has been limited [12]. Therefore, to ensure representation from all WHO regions of the world, and ensure representation from low- and middle-income countries, researchers and research institutions have been approached to invite them to participate. Members of the CATs and the International PPA Speech and Language Therapy/Pathology network have agreed to participate as collaborators in the study. Collaborators across 14 countries have agreed to participate and have ethical approval including UK, Chile, Canada, Australia, India, Germany, Portugal, Italy, Israel, Spain, USA, Netherlands, Norway and Brazil. Having agreed to participate, a manual and slide deck will be shared with collaborators instructing them on the methodology of how to run the groups (remotely where required by COVID-19 restrictions) in their respective countries. Collaborators have been asked to seek ethical approval or equivalent from their relevant institution and to translate materials where required (see ethical approvals listed).

Recruitment of participants: Collaborators will approach people with PPA, and their families via networks known to them in each country and invite them, via email, to participate in a one-off group meeting. Participants will complete consent procedures required by each collaborator's institution.

Procedures: Collaborators will collate demographic data from participants on age, sex, type of PPA (including nfvPPA, svPPA, lvPPA and no specific variant of PPA diagnosed) and time since diagnosis. We anticipate this will include both mildly and more severely affected people with PPA. Meetings will be held either online, via video conferencing, or in person, depending

For peer review only

on the current COVID-19 restrictions and ethical approval guidance within the relevant country. Participants with PPA will attend one meeting and family members will attend a different meeting. Participants with PPA will be asked, “What would you most like to change about your communication and the way PPA affects your life?”. Family members will be asked,“What would you most like to change about your family member’s communication and the way PPA affects your life?”. Following NGT [32,33] methods the participants will be invited to generate a list of items in response to the question, which will be shared in a round robin style with the remaining group members. Having collated a list of ideas, participants will be asked to identify their top three items in order of priority. A copy of the study manual is available in the supplementary material.

Analysis: Demographic data will be anonymised, and descriptive statistics comprising mean values will be calculated. The group facilitator (the collaborator in each country) will weight each answer (top items will be weighted with a 3, second with a 2 and third with a 1), and aggregate individual scores to produce a final list of results, identifying the top three rated items for each group (people with PPA and their families) in each country. Anonymised results collected from different countries will be shared with the lead author (AV), who will aggregate items in line with NGT methodology [32,33] and produce an overall list of results, and demographic data. Results will consider both an overall PPA outcomes list of results, and those that align with particular PPA variants.

Ethical approval: Ethical has been sought by each collaborator at their participating institutions and participants will be consented based on local guidance. The Stage 2 UK collaboration is being undertaken as part of the Rare Dementia Support (RDS) Impact Study which received approval from the UCL Research Ethics Committee (8545/004: Rare Dementia Support Impact Study). All consent sessions will be video recorded, in line with the approved procedure outlined in the RDS Impact study protocol (Brotherhood et al., 2020). For the collaboration with Dr Carolina Mendez in Chile, ethical approval has been granted by the Pontificia Universidad Catolixa de Chile Ethics Committee, ID no. 190510002. For the collaboration with Dr Regina Jokel in Canada, ethical approval has been granted by Baycrest, Research Ethics Board REB 22-37. For the collaboration with Dr Jade Cartwright and Dr Cathy Taylor-Rubin in Australia ethical approval has been granted by Southeastern Sydney Local Health District HREC 2022/ETH02740. For the collaboration with Dr Avanthi Paplikar in India ethical approval has been granted by the Bangalore Speech and Hearing Research Foundation. For the

For peer review only

collaboration with Prof Marcus Meinzer, Anna Rysop and Nina Unger in Germany ethical approval has been granted by Griefswald University Ethics Committee, Germany, Reference BB 130/22. For the collaboration with De Ines Cadorio in Portugal ethical approval has been granted by Ethics Committee of the University Fernando Pessoa Prot n. 50/C.E> del 28/02/22. For the collaboration with Dr Petronilla Battista in Italy, ethical approval was granted by the ethics Committee of the IRCCS Giovanni Paolo II Bari, Prot. n. 80/CE Maugeri on 17/02/2022. For the collaboration with Dr Adi Lifshitz Ben basat and Hagit Bar-Zeev in Israel ethical approval was granted by the Ariel University Ethics Board, Israel. For the collaboration with Dr Maya Henry and Carly Milanski in America ethical approval was given by the Office of Research Support and Compliance and the University of Texas at Austin's Institutional Review Board IRB ID STUDY00000717-MOD06. For the collaboration with Dr Sandra Weilart, Dr Lize Jiskoot, Janna Vanegmond, Heleen Hendriksen and Antoinette Keulen in the Netherlands ethical approval was granted by Amsterdam UMC under the number 2023.0098. For the collaboration with Dr Monica Norvik in Norway ethical approval was granted by the Norwegian Agency for Shared Services in Education and Research (SIKT) ref: 865145. For collaboration with Maria Isabel d'Avila Freitas in Brazil ethical approval was granted by Hospital of Clinics- Faculty of medicine – Uni of Sao Paulo (USP) ref: 4.142.664.

Data collected from both Stage 1 & 2 will be presented to the project PPI group for analysis and discussion to agree the key constructs that should inform discussion at the final consensus group. Given that several members of the PPI group have communication difficulties, information will be presented in an accessible format, using images and aphasia accessible written and spoken language. Gaps in the data and reasons for this will be considered and will inform prioritisation of information for the final consensus meeting.

Stage 3: COS-PPA Consensus work to identify and agree core outcomes and measurement sets

Consensus work to agree a final COS-PPA with the team of international cross-disciplinary researchers will be undertaken using Delphi consensus methods [32]. This will include identifying and agreeing the core outcome measurement instruments for each of key constructs identified in Stages 1 and 2. Stage 3 work has been granted ethical approved by UCL Language and Cognition Department Ethics Committee, Project ID LCD-2023-06.

Participants: Participants will be recruited via international networks of researchers working in the field of PPA intervention research. Given researchers in this field may include speech and language researchers, neuropsychology, psychology, neurology, occupational therapy and other groups, it will be essential to explore networks within and outside of the International PPA Speech and language Therapy / Pathology network and CATS. Researchers and authors of studies identified in the Stage 1 systematic review will be contacted, in addition to contacting researchers via additional networks including the International Society of Fronto-Temporal Dementia (ISFTD). The authors will purposefully aim to recruit researchers across a range of countries, without overly biasing representation from one country.

Procedures: Emails will be sent inviting researchers to participate in the study. Should they agree by return of email, they will be sent a link to complete an online consent form and brief survey collecting demographic information (including affiliation, professional background, research interests, country of current work, qualification, number of PPA participants seen as part of research, languages in which research undertaken) and availability to attend a meeting hosted on a video conferencing platform.

Prior to attending video conferencing meetings, researchers will be asked to complete an online vote rating the importance of outcome constructs (selected based on data collated in Stage 2 of the study). Researchers will rate each construct on a scale of importance with 9 being the most important and 1 being the least important. Rankings of 7-9 indicate critical importance, 4-6 outcomes that are important but not critical, while ratings of 1-3 are of limited importance using the Grading of Recommendations Assessment, Development and Evaluations (GRADE) scale [34]. Researchers will be invited to put other constructs forward that they think are important but had not been included in the survey. They will also be asked about which constructs require different measurements for individual PPA variants and which do not. Results of the first round of voting will be re-disseminated and researchers will be asked to re-vote on the importance of these constructs.

Only researchers who have completed the online ratings will be invited to participate in online meetings. At least two online meetings, hosted on Zoom, will be held to capture researchers across different zones. Meetings will be facilitated by an independent facilitator, not affiliated with research in this field. For each construct researchers will be presented with measures identified in the Stage 1 systematic review that measure this construct. Researchers will be provided with a description of each measure, including its psychometric properties and

For peer review only

languages in which it is available, and will then be asked to vote on which measures they feel best address the construct. Measures will not be excluded if there is no psychometric data available. Researchers will also be asked to put forward any alternative measures. Results of the first round of voting, including any new suggestions will be disseminated to the researchers via email. They will then be invited to re-vote, to identify which measure would be best as a core outcome measure for each construct.

Analysis: Demographic information will be analysed using descriptive statistics. Anonymised voting data collected in the modified Delphi consensus study will be aggregated and data italicised for presentation.

Dissemination:

Work undertaken at Stages 1, 2 and 3 will be published in open-access peer reviewed journal articles and presented at international scientific conferences. The results will additionally be published in clinical practice magazines in the UK, the Royal College of Speech and Language Therapy Bulletin magazine and Practical Neurology, and equivalent non-UK practice outlets identified by collaborators in relevant participating countries. The work will also be submitted to the World Health Organization's Global Observatory Knowledge Exchange Platform [35], a platform designed specifically to share knowledge about dementia research in response to the Global Action Plan on the Public Health Response to Dementia 2017-2025 [36].

Prior to publication, findings from the Stage 2 work to identify the most important outcomes for people with PPA and their families will be shared with participating international collaborators in the form of a draft manuscript. They will be invited to be co-authors, and to comment on the manuscript prior to submission to a peer reviewed journal. Additionally, it will be suggested that they share co-produced accessible materials with local participants.

Consensus work undertaken at Stage 3 will be shared by email with participants. Researchers participating in the study will be invited, at the outset, to collaborate on a peer reviewed manuscript, as co-authors. Having remained involved in the consensus process, researchers will be reminded of this and invited to collaborate and comment on a draft manuscript. As co-authors on the paper, they will assist in the dissemination of the results as well as receiving continuing updates on the research study.

For peer review only

The PPI contributors and the study steering group will be acknowledged in all submitted manuscripts and scientific conference presentations.

Discussion:

This protocol describes the development of a COS for PPA (COS-PPA) following three stages to identify the current measures used in the intervention research literature, what is important to people with PPA and their families internationally and finally a modified-Delphi consensus exercise to agree the COS. There is currently no published COS for PPA, and existing COSs for Dementia [14,15] and non-progressive aphasia [16,17] do not address the unique and specific needs of this group. The current research literature on PPA interventions uses a variety of outcomes [8], limiting comparability across studies and moreover does not address what is important to people with PPA and their families. Identifying measures that reflect the needs of people with all PPA variants will be challenging. It is essential that we understand the priorities of this seldom-heard group of people with speech, language, and communication difficulties and that we embed these in our research design. Using a COS could allow for combination of datasets, which, in the current setting of smaller studies would increase the power of the intervention studies and increase our understanding of efficacy of interventions for PPA. Given our collaborations internationally, the intention is for this COS to be used internationally, thus the dissemination plan includes both publications in peer reviewed journals and international dissemination platforms. We anticipate that there will be few measures available that have been translated into different languages. In fact, it is likely that there may be no suitable measures to address all the identified constructs and that are validated or developed for use with people with PPA. This highlights the potential for this study to inform and help prioritise more research to explore how PPA presents differently across languages, in both monolingual and multilingual speakers. Our ambition is to support the development of new measures for people with PPA, and their translation and validation across different languages. Given the differences in languages, it is anticipated that these measures may not necessarily focus on linguistic performance, but on patient reported outcome measures. As an example, a communication function such as coherent and connected propositional speech is key to effective communication in English, Chinese or Turkish (etc) even though the specific linguistic vehicles may vary widely between languages. This in turn, will potentially have far-reaching implications beyond the scope of PPA, extending to people living with other dementia types, who also have speech, language, and communication needs such as Posterior Cortical Atrophy

For peer review only

[37], Frontotemporal Dementia, Young Onset Alzheimer's Disease, Typical Alzheimer's Disease and Lewy Body Dementia [38].

Abbreviations:

Trial Status:

Stage 1 Systematic review searches March 2023, data extraction May 2023

Stage 2 Nominal Groups with people with PPA July 2021-May 2023, Survey of Speech and Language Therapists June 2023

Stage 3 Modified Delphi Consensus methods July 2023-October 2023

Ethical approval:

All aspects of the study will be conducted according to the Declaration of Helsinki.

The Stage 2 UK collaboration is being undertaken as part of the Rare Dementia Support (RDS) Impact Study which received approval from the UCL Research Ethics Committee (8545/004: Rare Dementia Support Impact Study). All consent sessions will be video recorded, in line with the approved procedure outlined in the RDS Impact study protocol (Brotherhood et al., 2020). For the collaboration with Dr Carolina Mendez in Chile, ethical approval has been granted by the Pontificia Universidad Catolixa de Chile Ethics Committee, ID no. 190510002. For the collaboration with Dr Regina Jokel in Canada, ethical approval has been granted by Baycrest, Research Ethics Board REB 22-37. For the collaboration with Dr Jade Cartwright and Dr Cathy Taylor-Rubin in Australia ethical approval has been granted by Southeastern Sydney Local Health District HREC 2022/ETH02740. For the collaboration with Dr Avanthi Paplikar in India ethical approval has been granted by the Bangalore Speech and Hearing Research Foundation. For the collaboration with Prof Marcus Meinzer, Anna Rysop and Nina Unger in Germany ethical approval has been granted by Griefswald University Ethics Committee, Germany, Reference BB 130/22. For the collaboration with De Ines Cadorio in Portugal ethical approval has been granted by Ethics Committee of the University Fernando Pessoa Prot n. 50/C.E> del 28/02/22. For the collaboration with Dr Petronilla Battista in Italy, ethical approval was granted by the ethics Committee of the IRCCS Giovanni Paolo II Bari, Prot. n. 80/CE Maugeri on 17/02/2022. For the collaboration with Dr Adi Lifshitz Ben basat and Hagit Bar-Zeev in Israel ethical approval was granted by the Ariel University Ethics Board, Israel. For

For peer review only

the collaboration with Dr Maya Henry and Carly Milanski in America ethical approval was given by the Office of Research Support and Compliance and the University of Texas at Austin's Institutional Review Board IRB ID STUDY00000717-MOD06. For the collaboration with Dr Sandra Weilart, Dr Lize Jiskoot, Janna Vanegmond, Heleen Hendriksen and Antoinette Keulen in the Netherlands ethical approval was granted by Amsterdam UMC under the number 2023.0098. For the collaboration with Dr Monica Norvik in Norway ethical approval was granted by the Norwegian Agency for Shared Services in Education and Research (SIKT) ref: 865145. For collaboration with Maria Isabel d'Avila Freitas in Brazil ethical approval was granted by Hospital of Clinics- Faculty of medicine – Uni of Sao Paulo (USP) ref: 4.142.664.

Stage 3 work has been granted ethical approved by the Chairs of UCL Language and Cognition Department Ethics, Project ID LCD-2023-06. All participants involved will be asked to complete a written form consent before participation in the Delphi survey.

Funding sources:

This study was initially funded by a UCL Grand Challenge awarded to AV and CJH in 2020, and consequently by an NIHR Advanced Fellowship NIHR302240 to AV. Sarah J. Wallace is funded by a National Health and Medical Research Council (NHMRC) Investigator Grant (1175821). JW has received grant support from the Alzheimer's Society, Alzheimer's Research UK, the Royal National Institute for Deaf People (Discovery Grant G105_WARREN), the National Institute for Health Research University College London Hospitals Biomedical Research Centre and the National Brain Appeal (Frontotemporal Dementia Research Studentship in Memory of David Blechner).

Conflicts of interest:

All authors declare that there are no known conflicts of interest.

Author contributions:

AV is the chief investigator, she conceived the study, led the proposal and protocol development.

CJDH contributed to the study design and development of the proposal.

SW and DC contributed to the study design.

RV, MH and JDW contributed to finalising the proposal.

All authors have contributed to the manuscript preparation.

For peer review only

Acknowledgements:

The authors would like to acknowledge the invaluable assistance and advice of all the PPI contributors, the study steering group (Dr Paul Conroy, Dr Cath Mummery, Rosemary Townsend, Hannah Luff, Phillip Robinson and Samantha Stern). We would also like to thank all collaborators across all collaborating countries; Dr Carolina Mendez in Chile, Dr Regina Jokel in Canada, Dr Jade Cartwright and Dr Cathy Taylor-Rubin in Australia, Dr Avanthi Paplikar in India, Prof Marcus Meinzer, Anna Rysop and Nina Unger in, Dr Ines Cadorio in Portugal, Dr Petronilla Battista in Italy, Dr Adi Lifshitz Ben basat and Hagit Bar-Zeev in Israel, Dr Maya Henry and Carly Milanski in America, Dr Sandra Weilart, Dr Lize Jiskoot, Janna Vanegmond, Heleen Hendriksen and Antoinette Keulen in the Netherlands, Dr Monica Norvik in Norway, Maria Isabel d'Avila Freitas in Brazil, Dr Ibrahim Can Yasa in Turkey and Antoine Renard in France.

References:

1. Gorno-Tempini ML, Hillis AE, Weintraub S, Kertesz A, Mendez M, Cappa SF, Ogar JM, Rohrer JD, Black S, Boeve BF, Manes F. Classification of primary progressive aphasia and its variants. Neurology. 2011 Mar 15;76(11):1006-14.
2. Marshall CR, Hardy CJ, Volkmer A, Russell LL, Bond RL, Fletcher PD, Clark CN, Mummery CJ, Schott JM, Rossor MN, Fox NC. Primary progressive aphasia: a clinical approach. Journal of neurology. 2018 Jun;265:1474-90.
3. Ruksenaite J, Volkmer A, Jiang J, Johnson JC, Marshall CR, Warren JD, Hardy CJ. Primary progressive aphasia: toward a pathophysiological synthesis. Current neurology and neuroscience reports. 2021 Mar;21:1-2.
4. Carthery-Goulart MT, Silveira AD, Machado TH, Mansur LL, Parente MA, Senaha ML, Brucki SM, Nitrini R. Nonpharmacological interventions for cognitive impairments following primary progressive aphasia: A systematic review of the literature. Dementia & Neuropsychologia. 2013 Jan;7:122-31.
5. Cadório I, Lousada M, Martins P, Figueiredo D. Generalization and maintenance of treatment gains in primary progressive aphasia (PPA): A systematic review. International journal of language & communication disorders. 2017 Sep;52(5):543-60
6. Cotelli M, Manenti R, Ferrari C, Gobbi E, Macis A, Cappa SF. Effectiveness of language training and non-invasive brain stimulation on oral and written naming performance in

Primary Progressive Aphasia: A meta-analysis and systematic review. Neuroscience & Biobehavioral Reviews. 2020 Jan 1;108:498-525.

7. Nissim NR, Moberg PJ, Hamilton RH. Efficacy of noninvasive brain stimulation (tDCS or TMS) paired with language therapy in the treatment of primary progressive aphasia: an exploratory meta-analysis. Brain Sciences. 2020 Aug 28;10(9):597.
8. Volkmer A, Spector A, Meitanis V, Warren JD, Beeke S. Effects of functional communication interventions for people with primary progressive aphasia and their caregivers: A systematic review. Aging & mental health. 2020 Sep 1;24(9):1381-93.
9. Volkmer A, Walton H, Swinburn K, Spector A, Warren JD, Beeke S. Results from a randomised controlled pilot study of the Better Conversations with Primary Progressive Aphasia (BCPPA) communication partner training program for people with PPA and their communication partners. Pilot and Feasibility Studies. 2023 May 23;9(1):87.
10. Williamson PR, Altman DG, Bagley H, Barnes KL, Blazeby JM, Brookes ST, Clarke M, Gargon E, Gorst S, Harman N, Kirkham JJ. The COMET handbook: version 1.0. Trials. 2017 Jun;18:1-50.
11. Williamson PR, Altman DG, Blazeby JM, Clarke M, Devane D, Gargon E, Tugwell P. Developing core outcome sets for clinical trials: issues to consider. Trials. 2012 Dec;13(1):1-8.
12. Kirkham JJ, Davis K, Altman DG, Blazeby JM, Clarke M, Tunis S, Williamson PR. Core outcome Set-STAndards for development: the COS-STAD recommendations. PLoS medicine. 2017 Nov 16;14(11):e1002447.
13. Harding, A. J., Morbey, H., Ahmed, F., Opdebeeck, C., Wang, Y. Y., Williamson, P., ... & Reilly, S. (2018). Developing a core outcome set for people living with dementia at home in their neighbourhoods and communities: study protocol for use in the evaluation of non-pharmacological community-based health and social care interventions. Trials, 19, 1-13.
14. Harding, A. J., Morbey, H., Ahmed, F., Opdebeeck, C., Lasrado, R., Williamson, P. R., ... & Reilly, S. T. (2019). What is important to people living with dementia?: the 'long-list'of outcome items in the development of a core outcome set for use in the evaluation of non-pharmacological community-based health and social care interventions. BMC geriatrics, 19(1), 1-12.
15. Reilly, S. T., Harding, A. J., Morbey, H., Ahmed, F., Williamson, P. R., Swarbrick, C., ... & Keady, J. (2020). What is important to people with dementia living at home? A set of core outcome items for use in the evaluation of non-pharmacological community-based health and social care interventions. Age and ageing, 49(4), 664-671.

For peer review only - http://bmjopen.bmj.com/site/about/guidelines.xhtml

16. Wallace, S. J., Worrall, L., Rose, T., Le Dorze, G., Breitenstein, C., Hilari, K., ... & Webster, J. (2019). A core outcome set for aphasia treatment research: The ROMA consensus statement. International journal of stroke, 14(2), 180-185.
17. Wallace, S. J., Worrall, L., Rose, T. A., Alyahya, R. S., Babbitt, E., Beeke, S., ... & Dorze, G. L. (2022). Measuring communication as a core outcome in aphasia trials: Results of the ROMA-2 international core outcome set development meeting. International Journal of Language & Communication Disorders.
18. Loizidou M, Brotherhood E, Harding E, Crutch S, Warren JD, Hardy CJ, Volkmer A. 'Like going into a chocolate shop, blindfolded': What do people with primary progressive aphasia want from speech and language therapy?. International Journal of Language & Communication Disorders. 2022 Nov 30.
19. Hardy CJD, Taylor-Rubin C, Taylor B, Harding E, Gonzalez AS, Jiang J, Thompson L, Kingma R, Chokesuwattanaskul A, Walker F, Barker S, Brotherhood E, Waddington C, Wood O, Zimmermann N, Kupeli N, Yong KXX, Camic PM, Stott J, Marshall CR, Oxtoby NP, Rohrer JD, Volkmer A, Crutch SJ, Warren JD. Symptom-led staging for semantic and non-fluent/agrammatic variants of primary progressive aphasia. Alzheimers Dement. 2024 Jan;20(1):195-210. doi: 10.1002/alz.13415.
20. Belder CRS, Marshall CR, Jiang J, Mazzeo S, Chokesuwattanaskul A, Rohrer JD, Volkmer A, Hardy CJD, Warren JD. Primary progressive aphasia: six questions in search of an answer. J Neurol. 2024 Feb;271(2):1028-1046. doi: 10.1007/s00415-023-12030-4.
21. World Health Organisation's regions of the world https://www.who.int/about/who-we-are/regional-offices
22. Kirkham JJ, Gorst S, Altman DG, Blazeby JM, Clarke M, Tunis S, Williamson PR, COS-STAP Group Adrian Aldcroft Heather Bagley Bronwen Connolly Sarah Gorst Jamie J. Kirkham Tianjing Li Karen Matvienko-Sikar Victoria Thomas Sean Tunis Paula R. Williamson. Core outcome Set-STAndardised protocol items: the COS-STAP statement. Trials. 2019 Dec;20:1-7.
23. NIHR Patient and Public Involvement Resources (Viewed March 2023) https://www.nihr.ac.uk/documents/ppi-patient-and-public-involvement-resources-for-applicants-to-nihr-research-programmes/23437
24. Volkmer A, Broomfield K, editors. Seldom Heard Voices in Service User Involvement: The how and why of meaningful collaboration. J & R Press Limited; 2022.
25. Williamson, P.R., Altman, D.G., Bagley, H. *et al.* The COMET Handbook: version 1.0. *Trials* **18** (Suppl 3), 280 (2017). https://doi.org/10.1186/s13063-017-1978-4

26. Wauters, L. D., Croot, K., Dial, H. R., Duffy, J. R., Grasso, S. M., Kim, E., ... & Henry, M. L. (2023). Behavioral treatment for speech and language in primary progressive aphasia and primary progressive apraxia of speech: A systematic review. *Neuropsychology Review*, 1-42.
27. WHO International Classification of Functioning, Disability and Health (ICF) domains
28. Kertesz, A., Davidson, W., McCabe, P., Takagi, K., & Munoz, D. (2003). Primary progressive aphasia: diagnosis, varieties, evolution. *Journal of the International Neuropsychological Society*, *9*(5), 710-719.
29. Mesulam, M. M. (2001). Primary progressive aphasia. *Annals of neurology*, *49*(4), 425-432.
30. COSMIN guidance (Viewed March 2023) https://static-content.springer.com/esm/art%3A10.1186%2Fs13063-016-1555-2/MediaObjects/13063_2016_1555_MOESM2_ESM.pdf
31. Wallace SJ, Worrall L, Rose T, Le Dorze G, Cruice M, Isaksen J, Kong AP, Simmons-Mackie N, Scarinci N, Gauvreau CA. Which outcomes are most important to people with aphasia and their families? An international nominal group technique study framed within the ICF. Disability and rehabilitation. 2017 Jul 3;39(14):1364-79.
32. McMillan SS, King M, Tully MP. How to use the nominal group and Delphi techniques. International journal of clinical pharmacy. 2016 Jun;38:655-62.
33. McMillan SS, Kelly F, Sav A, Kendall E, King MA, Whitty JA, Wheeler AJ. Using the nominal group technique: how to analyse across multiple groups. Health Services and Outcomes Research Methodology. 2014 Sep;14:92-108.
34. Guyatt GH, Oxman AD, Vist GE, Kunz R, Falck-Ytter Y, Alonso-Coello P, et al. GRADE: an emerging consensus on rating quality of evidence and strength of recommendations. Bmj. 2008;336(7650):924-6.
35. World Health Organization's Global Observatory Knowledge Exchange Platform (Viewed March 2023) https://www.who.int/data/gho/data/themes/global-dementia-observatory-gdo
36. Global Action Plan on the Public Health Response to Dementia 2017-2025. (Viewed March 2023) https://www.who.int/publications/i/item/global-action-plan-on-the-public-health-response-to-dementia-2017---2025
37. Volkmer A, Farrington-Douglas C, Crutch S, Beeke S, Warren J, Yong K. Better conversations: a language and communication intervention for aphasia in posterior cortical atrophy. Neurocase. 2022 Jul 4;28(4):356-63.

For peer review only

38. Suárez-González A, Cassani A, Gopalan R, Stott J, Savage S. When it is not primary progressive aphasia: a scoping review of spoken language impairment in other neurodegenerative dementias. Alzheimer's & Dementia: Translational Research & Clinical Interventions. 2021;7(1):e12205.

For peer review only

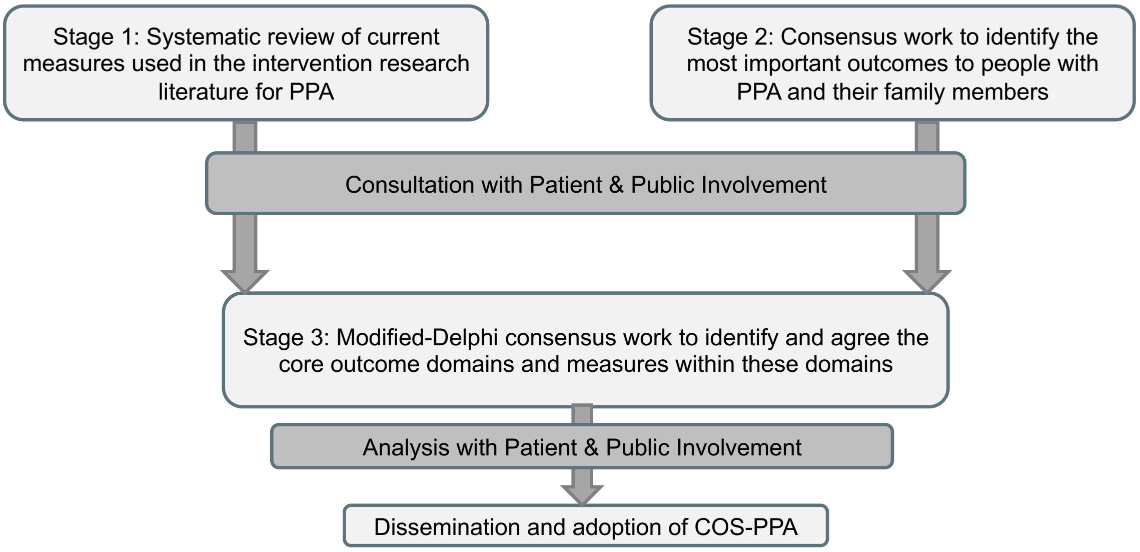

For peer review only

Core Outcome Set-STandards Protocol Items: The COS-STAP Statement Checklist

SECTION/TOPIC	ITEM No.	CHECKLIST ITEM	REPORTED ON PAGE NUMBER
TITLE/ABSTRACT			
Title	1a	Identify in the title that the paper describes the protocol for the planned development of a COS	1
Abstract	1b	Provide a structured abstract	2
INTRODUCTION			
Background and objectives	2a	Describe the background and explain the rationale for developing the COS, and identify the reasons why a COS is needed and the potential barriers to its implementation	3-4
	2b	Describe the specific objectives with reference to developing a COS	5
Scope	3a	Describe the health condition(s) and population(s) that will be covered by the COS	5
	3b	Describe the intervention(s) that will be covered by the COS	5
	3c	Describe the context of use for which the COS is to be applied	5
METHODS			
Stakeholders	4	Describe the stakeholder groups to be involved in the COS development process, the nature of and rationale for their involvement and also how the individuals will be identified; this should cover involvement both as members of the research team and as participants in the study	6,7,8
Information sources	5a	Describe the information sources that will be used to identify the list of outcomes. Outline the methods or reference other protocols/papers	3-4, 8-13
	5b	Describe how outcomes may be dropped/combined, with reasons	8-13
Consensus process	6	Describe the plans for how the consensus process will be undertaken	10-13
Consensus definition	7a	Describe the consensus definition	12
	7b	Describe the procedure for determining how outcomes will be added/combined/dropped from consideration during the consensus process	12
ANALYSIS			
Outcome scoring/feedback	8	Describe how outcomes will be scored and summarised, describe how participants will receive feedback during the consensus process	12-13
Missing data	9	Describe how missing data will be handled during the consensus process	10-13
ETHICS and DISSEMINATION			
Ethics approval/informed consent	10	Describe any plans for obtaining research ethics committee/institutional review board approval in relation to the consensus process and describe how informed consent will be obtained (if relevant)	15

For peer review only

Dissemination	11	Describe any plans to communicate the results to study participants and COS users, inclusive of methods and timing of dissemination	13
ADMINISTRATIVE INFORMATION			
Funders	12	Describe sources of funding, role of funders	15
Conflicts of interest	13	Describe any potential con	15

From: Kirkham JJ, Gorst S, Altman DG, et al. (2019) Core Outcome Set-STAndardised Protocol Items: the COS-STAP Statement. Trials 20, 116. https://doi.org/10.1186/s13063-019-3230-x

For peer review only

IMPROVING

PRIMARY PROGRESSIVE APHASIA: DEVELOPMENT OF A CORE OUTCOME SET

NOMINAL GROUP TECHNIQUE (NGT)

MANUAL

CHIEF INVESTIGATOR:

PI: DR ANNA VOLKMER

LANGUAGE AND COGNITION, UCL

ADAPTED WITH PERMISSION FROM DR SARAH WALLACE

For peer review only - http://bmjopen.bmj.com/site/about/guidelines.xhtml

HOW TO USE THIS MANUAL

This manual contains the information that you will need to run your nominal group/s.

Throughout the manual you will see the following symbols:

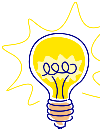

The 'light bulb' symbol will alert you to tips or extra information.

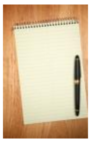

The 'paper and pen' symbol indicates that there is a relevant template in the appendix. You **may need to translate these documents or modify them** to reflect your language/ location or details specific to your site.

Any questions or comments about this manual or study can be directed to

Anna Volkmer

For peer review only - http://bmjopen.bmj.com/site/about/guidelines.xhtml

TABLE OF CONTENTS

HOW TO USE THIS MANUAL **2**

1. EXECUTIVE SUMMARY .... ERROR! BOOKMARK NOT DEFINED.

2. PROJECT OVERVIEW **5**

2.1 PROJECT AIM 5

2.2 BACKGROUND 5

2.3 PROJECT OVERVIEW 6

2.4 STUDY 1A: NOMINAL GROUP TECHNIQUE (NGT) 6

2.4.1 Objectives *6*

2.4.2 NGT overview *7*

2.4.3 Stages of the NGT *7*

2.4.4 Benefits of the NGT *8*

3. ETHICAL APPROVAL **8**

4. PARTICIPANTS **8**

4.1 ELIGIBILITY AND SAMPLING 8

4.2 GROUP COMPOSITION 10

5. RECRUITMENT **10**

6. VENUE AND RESOURCES **11**

6.1 VENUE 11

6.2 EQUIPMENT 11

7. PROCEDURES FOR RUNNING NOMINAL GROUPS **12**

9. PARTICIPANT FOLLOW-UP .... ERROR! BOOKMARK NOT DEFINED.

10. CONTACT DETAILS **17**

11. APPENDIX .... ERROR! BOOKMARK NOT DEFINED.

B. Slides and Nominal question .... **Error! Bookmark not defined.**

C. Participant information sheet .... **Error! Bookmark not defined.**

E. Participant consent form .... **Error! Bookmark not defined.**

G. Participant demographics .... **Error! Bookmark not defined.**

H. participant response sheet .... **Error! Bookmark not defined.**

12. REFERENCES **17**

For peer review only - http://bmjopen.bmj.com/site/about/guidelines.xhtml

For peer review only

For peer review only - http://bmjopen.bmj.com/site/about/guidelines.xhtml

1. PROJECT OVERVIEW

1.1 PROJECT AIM

To develop a core outcome set for research on intervention with people with PPA.

1.2 BACKGROUND

There is currently no curative treatment for Primary Progressive Aphasia (PPA), a language led dementia that progresses inexorably over time. Symptomatic pharmacological therapies have also not shown any evidence of effectiveness. Speech and language therapists and neuroscientists across the world have, however, worked for many years on tailored programmes for such people with PPA, and multiple interventions have emerged. Yet, research examining the effectiveness of these lack rigour, with small sample sizes and a lack of consistency in outcome measures posing limitations to the generalisability of the work. In this international cross-disciplinary collaboration we propose the development of a core set of outcome measures for researchers in the field of PPA interventions. This is extremely important in allowing this field of intervention research to develop, improving the rigour and impact of the work being undertaken. This will provide benefits for individuals with PPA worldwide, increasing access to interventions that can maintain communication, relationships and indepdenendence.

The COMET (Core Outcome Measures in Effectiveness Trials) initiative seeks to connect people interested in the development of core outcome sets. The COMET website houses a database (see http://www.comet-initiative.org) which currently contains 196 references of planned, ongoing and completed work on core outcome sets. OMERACT (Outcome Measures for Rheumatology Clinical Trials) is perhaps the best known initiative for the development of core outcome sets. OMERACT has used an iterative consensus process to develop a core outcome set for use in rheumatology clinical trials. It has a great focus on stakeholder consultation, with consumer, research, clinical and industry stakeholders participating in development process (Kirwan et al., 2003; Kvien & Heiberg, 2003; Quest et al., 2003). There is growing acknowledgement of the benefits of core outcome sets for research. Core sets have been developed or are being developed in over 50 fields including chronic pain (Dworkin et

For peer review only - http://bmjopen.bmj.com/site/about/guidelines.xhtml

al., 2005; McGrath et al., 2008; Turk et al., 2008), systemic sclerosis (Khanna, 2008; Khanna et al., 2008), childhood asthma (Sinha et al., 2012) and Eczema (Schmitt, Langan, Stamm, Williams, & Harmonizing Outcome Measurements in Eczema Delphi, 2011).

1.3 PROJECT OVERVIEW

This study will comprise a Nominal Group Technique (NGT) protocol previously devised by our collaborator, Dr Sarah Wallace, modifed to meet the needs of people with PPA. We will lead a team of international collaborators to hold meetings in countries identified through our networks of collaborators. Meetings will be held remotely where required by COVID-19 restrictions.

Participants and recruitment: Stakeholders will be purposivefully invited from third sector organisations, clinical and research networks, to represent people with PPA, their families, clinical speech and language therapists, neurologists and neuropsychologists. Meetings with people with PPA will be held separately from their family members and will therefore not exceed more than 4-6 participants to ensure the facilitator will be able to support participants' communication needs.

Analysis: Data will be analysed using aggregation methods consistent with NGT methodology.

1.4 STUDY 1A: NOMINAL GROUP TECHNIQUE (NGT)

1.4.1 OBJECTIVES

1. To identify outcomes of importance to people with PPA and their family and friends from an international sample.

For peer review only - http://bmjopen.bmj.com/site/about/guidelines.xhtml

2. To prioritise outcomes of importance to people with PPA and their family and friends from an international sample.

3. To categorise outcomes of importance into outcome domains (using World Health Organization (WHO) International Classification of Functioning, Disability and Health (ICF) domains (World Health Organization., 2001)).

4. To examine similarities and differences in outcomes of importance across stakeholder and country groups.

5. To determine which outcome domains have the highest representation within and across stakeholder and country groups.

6. To identify the ultimate desired outcome of speech and language therapy interventions according to participants with PPA and their family and friends.

1.4.2 NGT OVERVIEW

NGT is a structured group decision-making technique. In this technique a small group of participants are asked to respond to a question posed by a group facilitator. Participants are then asked to rank or prioritise these responses. The individual votes are then tallied to identify the ideas that are rated highest by the group as a whole. The NGT process encourages the participation of all group members and results in a set of prioritised responses (Delbecq et al., 1975). It is widely recognised as an effective method of gaining group consensus (Allen, Dyas, & Jones, 2004; Harvey & Holmes, 2012).

1.4.3 STAGES OF THE NGT

The NGT is divided into the following stages:

- Welcome and introduction: Purpose of session, rules and structure.
- Stage 1: Individual responses are collected.
- Stage 2: Individual responses are clarified and similar responses are grouped.
- Stage 3: Participants rank their top three responses in terms of personal importance.
- Responses are calculated and shared with the group.

For peer review only - http://bmjopen.bmj.com/site/about/guidelines.xhtml

- Thanks and close.

1.4.4 BENEFITS OF THE NGT

NGT has been previously used in the development of core outcome sets as a means of achieving consensus on outcomes, outcome domains and outcome instruments for inclusion in core sets (Douglas et al., 2009; Heiligenhaus et al., 2012; Khanna et al., 2008; Lamb et al., 2005).

NGT is suited to use with people with communication disability as it inherently supports communication through a structured 'round-robin' process of idea presentation which allows equal participation. The technique also encourages 'hitchhiking' (the stimulation of ideas in response to other group member responses) which increases opportunities for participation (Delbecq et al., 1975).

The technique has been used successfully with people with aphasia (Dorze, Julien, Brassard, Durocher, & Boivin, 1994; Garcia, Laroche, & Barrette, 2002; Lomas et al., 1989; Lomas, Pickard, & Mohide, 1987) and people with traumatic brain injury and associated communication disability (Larkins et al., 2004).

NGT is a cost and time effective method of data collection as the results are immediately available and no transcription is required.

2. ETHICAL APPROVAL

Ethical approval in the UK is part of the IMPACT study, UCL.

3. PARTICIPANTS

The NGT study has two participant groups: 1) people with PPA and 2) the family and friends of people with PPA and 3) bereaved family and friends of people with PPA.

3.1 ELIGIBILITY AND SAMPLING

People with PPA

Inclusion criteria:

For peer review only

- Aged 18 years or over.
- Diagnosis of PPA
- Ability to participate in the nominal group technique (NGT) process (as judged by an SLT).
- Living in the community.

Exclusion criteria:

- Comorbid significant health or mental health impairments (e.g. stroke or severe depression)

Sampling: Maximum variation sampling.

Sampling variables:

- Age
    - <100 years
    - >18 years
- Variant
    - Logopenic
    - Semantic
    - Non-fluent
- Gender
    - Male
    - Female
- World region
    - Africa
    - Asia
    - Australia
    - The Americas
    - Europe

Family and friends of people with PPA

Selection criteria:

- As above, except not diagnosed with PPA

Sampling: Convenience sampling

For peer review only - http://bmjopen.bmj.com/site/about/guidelines.xhtml

3.2 GROUP COMPOSITION

There should be a maximum 5 people per nominal group. Site co-ordinators should aim for at least one group of people with PPA and one group of family and friends.

For groups of people with PPA, try to ensure that as many of the following variables as possible are represented:

Gender	variant	Time post-onset	Age
Male	semantic	< 1 year	< 65 years
Female	logopenic	> 1 year	> 65 years
	Non-fluent		

4. RECRUITMENT

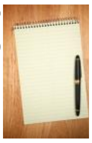

It is suggested that the onsite speech and language therapist/pathologist should recruit people with PPA and their family and friends through local avenues, such as email discussion groups, newsletters and community groups.

Interested participants should be provided with a more detailed information sheet (to be produced locally).

People with PPA who agree to participate in this study should also be asked to nominate a family member or friend to participate.

Separate nominal groups should be held for people with PPA and family/friend groups.

One or more groups may be held for each participant group. Each nominal group should have a maximum of 5 participants.

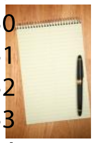

Demographic details should be recorded for each participant comprising diagnosis, time since onset, gender, age.

For peer review only - http://bmjopen.bmj.com/site/about/guidelines.xhtml

5. VENUE AND RESOURCES

5.1 VENUE

The nominal groups will be run online on zoom. Prior to facilitating the group the following should be clarified.

- Are clients able to access zoom with support as required?
- Can keywords can be written/typed to help facilitate the comprehension of people with PPA?

5.2 EQUIPMENT

To run your nominal group you will need:

- Pens and paper (for participants)
- Powerpoint slides pre-prepared with key questions/ headings
- Communication supports for participants e.g. communication (picture) boards
- Video recording facilities via zoom
- Some may need help from carers/ family members – to assist with collecting demographic details and recording participant responses.

For peer review only

For peer review only - http://bmjopen.bmj.com/site/about/guidelines.xhtml

6. PROCEDURES FOR RUNNING NOMINAL GROUPS

Each nominal group should have a maximum of 5 participants. People with PPA may have family member/friend present to support them if they desire. You may wish to have additional helpers to assist with collecting demographic information, write field notes and to help people with PPA to communicate their responses.

BEFORE THE DAY OF THE GROUP

Assign each participant a number

Each participant should be assigned a participant number using the following format:

Country	Stakeholder group	Group number	Number
Australia – AU<br>Canada – CA<br>United States – US<br>Israel– IL<br>Singapore – SP<br>India- In<br>Chile- Ch<br>Germany- Gr<br>United Kingdon- UK<br>Norway-Nw<br>Turkey- Tk<br>Iceland- IC	People with PPA – PWPPA<br>Family and friends - FF	If you have more than 1 nominal group for each stakeholder type, use a group number	Give each participant a number

e.g. AU-PWA-1-1

AU-PWA-1-2

For peer review only - http://bmjopen.bmj.com/site/about/guidelines.xhtml

ON THE DAY OF THE GROUP

Before the participants arrive

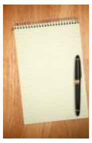

Consent will be sought prior to the group.

Meet prior to the NGT meeting/Email/ post the information sheet, consent form, question and response sheet and demographic information form for each participant.

Introduction (10 minutes)

Welcome the participants and provide a brief explanation of the purpose of the study and an outline of the procedure for the group using the following scripts. You may modify these scripts if necessary, however we are aiming for as much consistency across sites as possible. When presenting the introduction to people with PPA write key words on the whiteboard to maximise communicative access to the information.

For peer review only

Introduction Script – People with PPA

Thank you for coming today.

We are doing this study because we want to learn more about the outcomes (results from speech and language therapy) that are important to people with PPA.

We hope that this will help to improve PPA research and PPA services.

We are using a technique called a 'nominal group technique' to find out what is important to you. It allows everyone to contribute equally.

I will ask the group a question. Everyone will have time to think about their answers. We will then take turns to share our answers. Your answers don't have to be the ones you first thought of. If you think of something else you can add it. We will write the answers on the whiteboard. We will group similar answers together. You will have a chance to explain your responses. Then I will ask you to pick the three most important answers to you. We will help you to write the answers down on your sheet.

For peer review only - http://bmjopen.bmj.com/site/about/guidelines.xhtml

The information that you give today will be kept confidential and safe. The results may be published in a journal. Your name will not be used.

You will need to sign a consent form. It is your choice to participate in this study. It will take approximately an hour. You can stop anytime.

We will be video recording the group. This will be used check the information we collect. Are there any questions?

Introduction Script – Family and friends of people with PPA

Thank you for coming today.

This study aims to find out what outcomes are important to family and friends of people with PPA. We want to develop a set of tools to measure change following speech and language therapy. This is called a core outcome set. This will help us to improve PPA research. We hope it will also improve the services received by people living with PPA.

We will also be asking people with PPA, SLTs, policy makers and researchers what is important to them.

We are using a technique called a 'nominal group technique' to find out what is important to you. We are using this technique as it has been shown to allow everyone to contribute equally.

I will ask the group a question. Everyone will have time to think about their answers. We will then take turns to share our answers. Your answers don't have to be the ones you originally thought of. If you think of something else you can add it. We will write the answers on the whiteboard. We will group similar answers together. You will have a chance to explain or clarify your responses. Then I will ask you to pick the three most important answers to you and write them on your response sheet.

For peer review only - http://bmjopen.bmj.com/site/about/guidelines.xhtml

For peer review only

The information that you give today will be kept confidential and stored securely. The results may be published in a journal, but your name will not be used.

You will need to sign a consent form. It is your choice to participate in this study. You can stop anytime.

We will be video-recording the group. This will be used for data checking. It will take approximately 1 hour. Are there any questions?

Stage 1: Individual responses (25 minutes)

Start videoing. Inform participants that the video has been turned on and is now recording.

Read the following information to the group and present the nominal question verbally and in writing (write the question in large print on the whiteboard).

People with PPA	What would you most like to change about your communication and the way PPA affects your life?
Family and friends of people with PPA	What would you most like to change about your family member/friend's communication and the way PPA affects your life?

Allow a period of 5-10 minutes of/for quiet reflection. Ask group members to write down (if they are able) or think about as many responses as possible. If you have additional helpers in the room, they may assist people with PPA to write down their responses.

Give each group member the opportunity to verbally offer one response at a time in rounds, allowing all participants the opportunity to contribute their answers. Write responses onto a slide, which you are screen sharing with participants.

Encourage 'hitchhiking' – i.e. encourage group members to expand on the responses of other group members. Responses do not need to come from the individual's original list.

For peer review only - http://bmjopen.bmj.com/site/about/guidelines.xhtml

Stage 2: Clarification and consolidation (20 minutes)

Read through each response. Allow participants to explain or clarify their responses.

Group similar responses together. Duplicates may be combined or deleted.

Stage 3: Ranking responses (15 minutes)

Ask the participants to reflect on their own feelings and beliefs.

Ask participants to select the three outcomes that are most important to them from the group list.

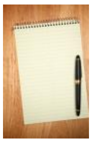

Ask participants to rank their top three most important outcomes, in order of personal importance. Enter this on the relevant slide in ranke order. Mark the column with the participant number.

AFTER THE GROUP

Thank participants.

Collate the final responses from each participant.

Note the amount of time that the session took.

7. DATA MANAGEMENT

The data will be stored safely on an encrypted laptop.

For peer review only - http://bmjopen.bmj.com/site/about/guidelines.xhtml

8. CONTACT DETAILS

Please direct any questions or correspondence to whomever is facilitating the group localy, or alternative Dr Anna Volkmer a.volkmer.15@ucl.ac.uk.

9. REFERENCES

Allen, J., Dyas, J., & Jones, M. (2004). Building consensus in health care: a guide to using the nominal group technique. [Research Support, Non-U.S. Gov't]. *Br J Community Nurs, 9*(3), 110-114.

Dorze, G. L., Julien, M., Brassard, C., Durocher, J., & Boivin, G. (1994). An analysis of the communication of adult residents of a long-term care hospital as perceived by their caregivers. *International Journal of Language & Communication Disorders, 29*(3), 241-268. doi: doi:10.3109/13682829409111610

Douglas, R. S., Tsirbas, A., Gordon, M., Lee, D., Khadavi, N., Garneau, H. C., . . . International Thyroid Eye Disease, S. (2009). Development of criteria for evaluating clinical response in thyroid eye disease using a modified Delphi technique. [Research Support, N.I.H., Extramural

Research Support, Non-U.S. Gov't]. *Arch Ophthalmol, 127*(9), 1155-1160. doi: 10.1001/archophthalmol.2009.232

Garcia, L. J., Laroche, C., & Barrette, J. (2002). Work integration issues go beyond the nature of the communication disorder. *Journal of Communication Disorders, 35*(2), 187-211. doi: http://dx.doi.org/10.1016/S0021-9924(02)00064-3

Harvey, N., & Holmes, C. A. (2012). Nominal group technique: an effective method for obtaining group consensus. [Research Support, Non-U.S. Gov't]. *Int J Nurs Pract, 18*(2), 188-194. doi: 10.1111/j.1440-172X.2012.02017.x

Heiligenhaus, A., Foeldvari, I., Edelsten, C., Smith, J. R., Saurenmann, R. K., Bodaghi, B., . . . Multinational Interdisciplinary Working Group for Uveitis in, C. (2012). Proposed

For peer review only - http://bmjopen.bmj.com/site/about/guidelines.xhtml

outcome measures for prospective clinical trials in juvenile idiopathic arthritis-associated uveitis: a consensus effort from the multinational interdisciplinary working group for uveitis in childhood. [Consensus Development Conference

Practice Guideline

Research Support, Non-U.S. Gov't

Review]. *Arthritis Care Res (Hoboken), 64*(9), 1365-1372. doi: 10.1002/acr.21674

Khanna, D., Lovell, D. J., Giannini, E., Clements, P. J., Merkel, P. A., Seibold, J. R., . . . Scleroderma Clinical Trials Consortium, c.-a. (2008). Development of a provisional core set of response measures for clinical trials of systemic sclerosis. [Research Support, N.I.H., Extramural

Research Support, Non-U.S. Gov't]. *Ann Rheum Dis, 67*(5), 703-709. doi: 10.1136/ard.2007.078923

Lamb, S. E., Jorstad-Stein, E. C., Hauer, K., Becker, C., Prevention of Falls Network, E., & Outcomes Consensus, G. (2005). Development of a common outcome data set for fall injury prevention trials: the Prevention of Falls Network Europe consensus. [Research Support, Non-U.S. Gov't]. *J Am Geriatr Soc, 53*(9), 1618-1622. doi: 10.1111/j.1532-5415.2005.53455.x

Lomas, J., Pickard, L., Bester, S., Elbard, H., Finlayson, A., & Zoghaib, C. (1989). The Communicative Effectiveness Index: Development and Psychometric Evaluation of a Functional Communication Measure for Adult Aphasia. *J Speech Hear Disord, 54*(1), 113-124.

Lomas, J., Pickard, L., & Mohide, A. (1987). Patient versus Clinician Item Generation for Quality-of-Life Measures: The Case of Language-Disabled Adults. *Medical Care, 25*(8), 764-769. doi: 10.2307/3765712

For peer review only - http://bmjopen.bmj.com/site/about/guidelines.xhtml
